# Accurate and reproducible enumeration of T-, B-, and NK lymphocytes using the BD FACSLyric 10-color system: A multisite clinical evaluation

**DOI:** 10.1371/journal.pone.0211207

**Published:** 2019-01-28

**Authors:** Imelda Omana-Zapata, Caren Mutschmann, John Schmitz, Sarah Gibson, Kevin Judge, Monika Aruda Indig, Beverly Lu, Doreen Taufman, Alan M. Sanfilippo, Wendy Shallenberger, Sharon Graminske, Rachel McLean, Rubal I. Hsen, Nicole d’Empaire, Kimberly Dean, Maurice O’Gorman

**Affiliations:** 1 BD Biosciences, San Jose, CA, United States of America; 2 SYNLAB Unweltinstitut GmbH, Berlin, Germany; 3 Department of Pathology and Laboratory Medicine, University of North Carolina, Chapel Hill, NC, United States of America; 4 Department of Pathology, University of Pittsburgh Medical Center Presbyterian, Pittsburgh, PA, United States of America; 5 Clinical Trial and Cellular Therapy Services, BloodCenter of Wisconsin, Milwaukee, WI, United States of America; 6 Department of Pathology and Laboratory Medicine, Children’s Hospital Los Angeles, Los Angeles, CA, United States of America; 7 BioCollections Inc., Miami, FL, United States of America; The Ohio State University, UNITED STATES

## Abstract

Clinical flow cytometry is a reliable methodology for whole blood cell phenotyping for different applications. The BD FACSLyric™ system comprises a flow cytometer available in different optical configurations, BD FACSuite™ Clinical software, and optional BD FACS™ Universal Loader. BD FACSuite Clinical software used with BD™ FC Beads and BD CS&T Beads enable universal setup for performance QC, instrument control, data acquisition/storage, online/offline data analysis, and instrument standardization. BD Biosciences sponsored the clinical evaluation of the BD FACSLyric 10-color configuration at seven clinical sites using delinked and de-identified blood specimens from HIV-infected and uninfected subjects to enumerate T-, B-, and NK-lymphocytes with the BD Multitest™ reagents (BD Multitest IMK kit and BD Multitest 6-color TBNK). Samples were analyzed on the BD FACSLyric system with BD FACSuite Clinical software, and on the BD FACSCanto™ II system with BD FACSCanto clinical software and BD FACS 7-Color Setup beads. For equivalency between methods, data (n = 362) were analyzed with Deming regression for absolute count and percentage of lymphocytes. Results gave R^2^ ≥0.98, with slope values ≥0.96, and slope ranges between 0.90–1.05. The percent (%) bias values were <10% for T- and NK cells and <15% for B- cells. The between-site (n = 4) total precision was tested for 5 days (2 runs/day), and gave %coefficient of variation below 10% for absolute cell counts. The stability claims were confirmed (n = 186) for the two BD Multitest reagents. The reference intervals were re-established in male and female adults (n = 134). The analysis by gender showed statistically significant differences for CD3^+^ and CD4^+^ T-cell counts and %CD4. In summary, the BD FACSLyric and the BD FACSCanto II systems generated comparable measurements of T-, B-, and NK-cells using BD Multitest assays.

## Introduction

Technical advances in flow cytometry have had a substantial impact in the understanding of the function and cell phenotyping of the T-, B-, and natural killer (NK) lymphocytes. These cells are involved in cell-mediated immunity in the human immune deficiency virus (HIV) infection, immune and auto-immune responses in cancer, bacterial and viral infections, asthma, and rheumatoid arthritis [1–5]. The T-, B-, and NK lymphocyte sub-sets can be identified with selective cell markers and reagents using flow cytometry methods, which are extensively used in different types of immune deficiencies [5–8]. For instance, enumeration of CD4 T-cell lymphocytes with flow cytometry and IVD reagents has been widely used in HIV-infected patients, continues to be used to assess the risk of opportunistic infections [8–10]. Clinical laboratories’ procedures are subject to continuous improvement in reducing errors and time to report results, to being flexible to adapt to changes, and to decreasing operational expenses without affecting throughput and quality of the results. Newer clinical analytical systems can simplify laboratory workflow, reduce the time or expertise required for analysis, and improve performance with high sensitivity and specificity, which enables improvement in sample testing throughput and cost reduction. Consistency in obtaining reproducible and accurate clinical results involves standardization of multiple factors across the laboratory testing continuum, including consistency in specimen collection and transportation, sample preparation, data acquisition, and reliable data analysis.

The BD FACSLyric system is for use as an *in vitro* diagnostic (IVD) device for identification and enumeration of human cell subsets. The system consists of a flow cytometer available in different optical configurations with BD FACSuite Clinical software, the optional BD FACS Universal Loader, and the BD FACSLink interface. BD FACSuite Clinical software is designed to be used with BD™ FC Beads and BD CS&T Beads, to support IVD universal setup (performance QC and instrument control), data acquisition, data storage, and online or offline data analysis. The BD FACSLink interface supports data transfer from BD FACSuite Clinical software to a laboratory information system (LIS).

The BD FACSLyric system has been designed to address the increasing complexity of clinical flow cytometry assays by simplifying instrument setup and by facilitating assay transfer across different instruments to improve efficiency and simplify the cell phenotyping workload. Clinical evaluation of the BD FACSLyric system involved four studies with the objectives of assessing different aspects of system performance. The hypotheses were that the BD FACSLyric system will generate results equivalent to the standard-of-care system, with an acceptable inter-laboratory variability, using BD Multitest reagents (BD Multitest IMK and BD Multitest 6-color TBNK). In addition, reagents tested in the BD FACSLyric system will validate results of the effects of specimen storage and stained lymphocyte storage and the reference intervals provided in the BD Multitest reagent package insert. The objectives of the clinical evaluation were: 1) to determine the difference between the BD FACSLyric 10-color system and the standard-of-care system for measuring absolute cell count and percentages of the T-, B-, and NK lymphocyte populations stained with BD Multitest reagents in BD Trucount™ tubes; 2) to evaluate the inter-laboratory BD FACSLyric system variability at four sites; 3) to evaluate robustness of the results of blood stored for 24 and 48 h and then stained at different time points with BD Multitest reagents; and 4) to define reference intervals for the T-, B-, and NK cells using the BD Multitest reagents, in a adult normal cohort of male and female subjects who were free of hematological abnormalities.

## Materials and methods

### Clinical sites and flow cytometry systems

The study sites were located in Berlin, Germany (1) and in the United States (6: Chapel Hill, North Carolina; Los Angeles and San Jose, California; Milwaukee, Wisconsin; Miami, Florida; and Pittsburgh, Pennsylvania). During this clinical evaluation, eight BD FACSLyric 10-color configuration flow cytometers with BD FACSuite Clinical software, BD CS&T beads, and BD FC Beads were used. The FC beads consist of dried polystyrene beads coupled to fluorophores in single-use tubes (FITC, PE, PerCP-Cy™5.5, PerCP, PE-Cy™7, APC and APC-Cy™7), and each tube requires rehydration with dilution buffer (phosphate buffered saline (PBS) with 0.1% sodium azide). In conjunction with the FACSuite clinical software and CS&T beads, the FC beads are able d to establish fluorescence compensation on the cytometer. The CS&T beads are used to measure the PMT voltages so the FC beads can determine the spillover values (SOVs) for the fluorescence compensation for each channel. Both, the FC beads and CS&T beads accurately sets the voltage and compensation for each of the fluorescence channels. The kit contains a bead lot file with the the lot number, expiry date and spectral overlap factors (SOFs) for each specific bead lot. The FACSuite Clinical software uses the information in the bead lot file to correct spillover values (SOVs) for differences between stained cells and the lot of beads.

**Fig 1:**
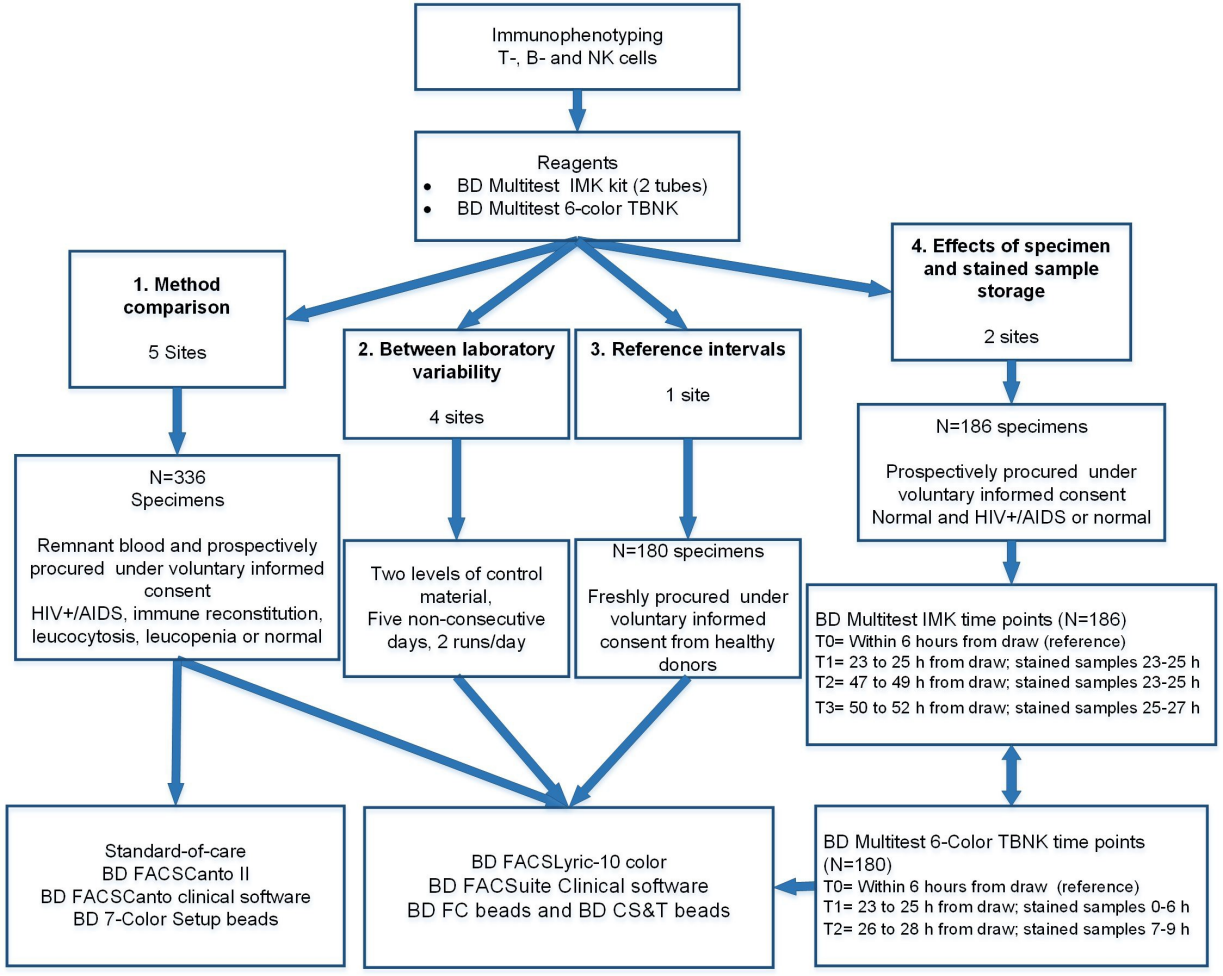
BD FACSLyric clinical evaluation flowchart. T-, B-, and NK cells were stained with BD Multitest reagents for testing of the 4 protocols depicted from the left to the right side: 1. Method comparison; 2. Inter-laboratory variability; 3. Determination of the reference intervals and 4. Evaluation of the specimen and stained sample storage. The summary for each protocol includes the number of specimens enrolled and cohort of origin, followed by the instrument(s) used for acquisition of the stained sample.

To show equivalence of performance in the method comparison study, the BD FACSLyric system was compared to the standard-of-care, the BD FACSCanto II system with BD FACSCanto clinical software and BD FACS 7-Color Setup Beads, for enumeration of T-, B-, and NK lymphocytes in whole blood remnant specimens. Remnant EDTA (K2 or K3) anti-coagulated specimens from healthy subjects or from HIV- infected, immune reconstitution, leucopenia or leukocytosis patients were assayed using BD Multitest reagents with BD Trucount tubes. For the inter-laboratory precision, stability and reference interval studies, the stained samples were tested only in the BD FACSLyric system. The inter-laboratory and system variability were addressed by testing the same sample at four clinical laboratories during five days, two runs per day. The stability evaluation of specimen and stained sample storage was conducted at two US study sites to show the effect of blood storage of <27 h and stained sample storage of <6 h for the BD Multitest 6-color TBNK kit, and blood storage of <51 h and stained sample storage of <24 h for the BD Multitest kit, using blood from HIV-infected or healthy subjects. The reference intervals were determined in ≥130 adult subjects at a single clinical site for both BD Multitest reagents. The subjects were hematologically healthy males and females of 18 years of age and older, who voluntary provided written informed consent prior to blood withdraw. See the study flow chart in **Fig 1**.

### Specimen procurement

Prospective collection of specimens under approved informed consent by the Medical College of Wisconsin/ Froedtert Hospital and the Chesapeake Institutional Review Boards. Protocols were reviewed and approved by iStar at USC Children's Hospital Los Angeles; the Western Institutional Review Board and the Biomedical IRB at UNC. For SYNLAB, the Ethics Commission, Berlin Medical Association indicated that the participating Physician has not duty to advise.

All venous whole blood was collected and anti-coagulated with EDTA (K2 and K3). Depending on the study, the specimens enrolled were either remnant specimens from routine laboratory testing that would be otherwise discarded, or prospectively procured from adult healthy subjects who voluntarily gave written informed consent. For the remnant specimens used for method comparison, samples were de-identified and the demographic information was not available. The prospectively procured venous blood anti-coagulated with EDTA was collected from a BD-qualified vendor blood donor pool using informed consent under good clinical practices [11] that were reviewed and approved by an institutional review board (IRB). For determining the reference intervals, individuals >18 years of age from a research donor pool at BloodCenter of Wisconsin were consented under an IRB approved, minimal risk protocol. The consented subjects provided a venous blood sample and limited information (age, gender, ethnicity, medical history and lifestyle activities), and their participation in the studies was limited to venous blood draw. After the site of skin puncture was closed, subjects were dismissed.

All specimens were de-identified and delinked from protected health information prior to enrollment, staining, and acquisition. Specimens were enrolled if they satisfied the inclusion criteria (for example, anti-coagulated with EDTA, enough volume, and enough time for staining); and were excluded if clotted, refrigerated, frozen, fixed or failed to comply with the protocol procedures (such as preparation and time testing requirements). The site’s Ethic Committee or IRB’s and BD Biosciences considered that the blood collection procedure and the use of remnant specimens present minimal risk to the subjects. Since there is no mandatory registration for minimal risk clinical studies, the clinical evaluation was not registered in the ClinicalTrials.gov. The results from testing were excluded from analysis if the testing process did not satisfy the protocol requirements and procedures.

### Study design

The study design for method comparison was based on the Clinical and Laboratory Standards Institute (CLSI) EP09-A3 guideline [12]. Remnant whole blood specimens from patients and healthy subjects were enrolled between December 2015 and March 2017 at four study sites for this study. The sample size was estimated to include a minimum of 240 specimens, exceeding the minimum recommended in the CLSI EP09-A3 guideline [12]. Samples were stained with the BD Multitest IMK kit 6-color TBNK reagents (BD Biosciences, San Jose, CA), and then analyzed on the BD FACSCanto II system with BD FACSCanto clinical software and on the BD FACSLyric system with BD FACSuite Clinical software (BD Biosciences, San Jose, CA).

The study design for inter-laboratory variability was based on CLSI EP5-A3 guidelines [13] and carried out on the BD FACSLyric system on five non-consecutive days, two runs per day, using a single lot of two levels of control material, CD-Chex Plus® CD4 Low and Normal Controls (Streck, USA). (See S1 File).

The study design for whole blood and stained sample stability was based on the CLSI EP25-A guideline [14]. The target sample size was a minimum of 90 prospectively procured specimens from healthy subjects and from patients with HIV-infected between January 2016 and February 2017 at two clinical sites. The specimens were stained and tested for the reference time point (T0) within six hours of draw. Blood was stored, stained, and tested on the BD FACSLyric system at the different time points for up to 53 hours to ensure complete testing (S2 File).

The study design for reference intervals was based on the CLSI EP28-A3 guideline [15]. The target sample size was a minimum of 120 specimens prospectively procured between June and October 2017 at a single study site from female and male healthy subjects free of hematological abnormalities. Samples were also stained with the BD Multitest IMK and 6-color TBNK reagents, and then analyzed on the BD FACSLyric system. (S3 File).

On each study day the testing was performed, the instrument setup was completed before running process controls. The BD FACSCanto II system was set up with BD FACS 7-color setup beads, and the BD FACSLyric system with BD CS&T beads. When process controls achieved acceptable results, the site proceeded to specimen enrollment. Each specimen was prepared for testing by staining the samples with the BD Multitest 6-color TBNK reagent with Trucount tubes and/or the BD Multitest IMK kit with Trucount tubes. The BD Multitest 6-color TBNK reagent (CD3 FITC / CD16 PE + CD56 PE / CD45 PerCP-Cy5.5 / CD4 PE-Cy7 / CD19 APC / CD8 APC-Cy7) comprised a single tube.

The BD Multitest IMK kit comprises two 4-color panels, each panel in one tube (CD3 FITC / CD8 PE / CD45 PerCP / CD4 APC and CD3 FITC / CD16 PE + CD56 PE / CD45 PerCP / CD19 APC). Briefly, to stain a sample, 50 μL whole blood was dispensed into a specimen ID-labeled Trucount tube, 20 μL respective reagent was added, and the tube was vortexed and incubated for 15 min in the dark. At the end of the incubation time, 450 μL of a BD proprietary buffered lysing solution containing <15% formaldehyde and <50% diethylene glycol (BD Biosciences, San Jose, CA) was added to each tube, and tubes were incubated again in the dark for 15 min. The stained samples were acquired using the corresponding flow cytometric system.

For the method comparison, four laboratories assigned different operators to the BD FACSCanto II and BD FACSLyric systems. At one site, however, the same operator prepared and acquired the samples on both systems.

### Data analysis

For method comparisons the data was grouped into either CD4 cell count bins (0 to <250; 250 to <500; 500 to 1000, and ≥1000 cells/μL), or %CD4 cell bins (0 to <20%; 20 to < 35%, and ≥35%). Data was analyzed for mean percent biases (%bias) between the BD FACSLyric and the standard-of-care of the absolute counts/μL and percentage of lymphocytes of the different lymphocyte subsets. Data was evaluated using weighted Deming regression fit for the absolute counts, regular Deming regression for the percentage of lymphocytes [16, 17] and Bland-Altman [18] methods for the absolute and percentage of cells for the T-, B-, and NK- lymphocytes. In addition, the method agreement and predicted %bias analyses were calculated at the clinically relevant CD4 T-cell count cutoff of 200 cells/μL.

The inter-laboratory variability analysis was carried out by calculating the coefficient of variation (CV) within 95% confidence intervals (95% CI) for absolute and percentage of cells for the T-, B-, and NK- lymphocytes across all sites (total precision) and per site.

The stability of blood and stained sample storage was assessed by calculating the bias between the results from specific time points for the respective reagent to the time point T0 (reference). The time points for each reagent were as follows:

- BD Multitest IMK kit: T0: Reference time point data acquired within 6 h from draw,.T1: Samples stained between 23 to 25 h from draw, data acquired between 23–25 h post-staining. T2: Samples stained between 47 to 49 h from draw, data acquired between 23–25 h. T3: Samples stained between 50 to 52 h from draw, data acquired between 25–27 h post-staining.
- BD Multitest 6-color TBNK: T0: Reference time point acquired within 6 hours from draw. T1: Samples stained between 23 to 25 h from draw, data acquired between 0–6 h post-staining. T2: Samples stained between 26 to 28 h from draw, data acquired between 7–9 h post-staining.

The reference intervals for the T-, B-, and NK cell populations were determined by calculating the 2.5 percentile and 97.5 percentile of the study population, and the 90% confidence interval with the upper and lower limits. Additional analyses were done per age group and gender partitions using ANOVA and Harris-Boyd methods recommended in the guideline [15].

For all the studies, the outlier values from the study were investigated and included in the analysis if there were no valid reasons for exclusion.

## Results

### Method comparison and inter-laboratory variability

**Fig 2:**
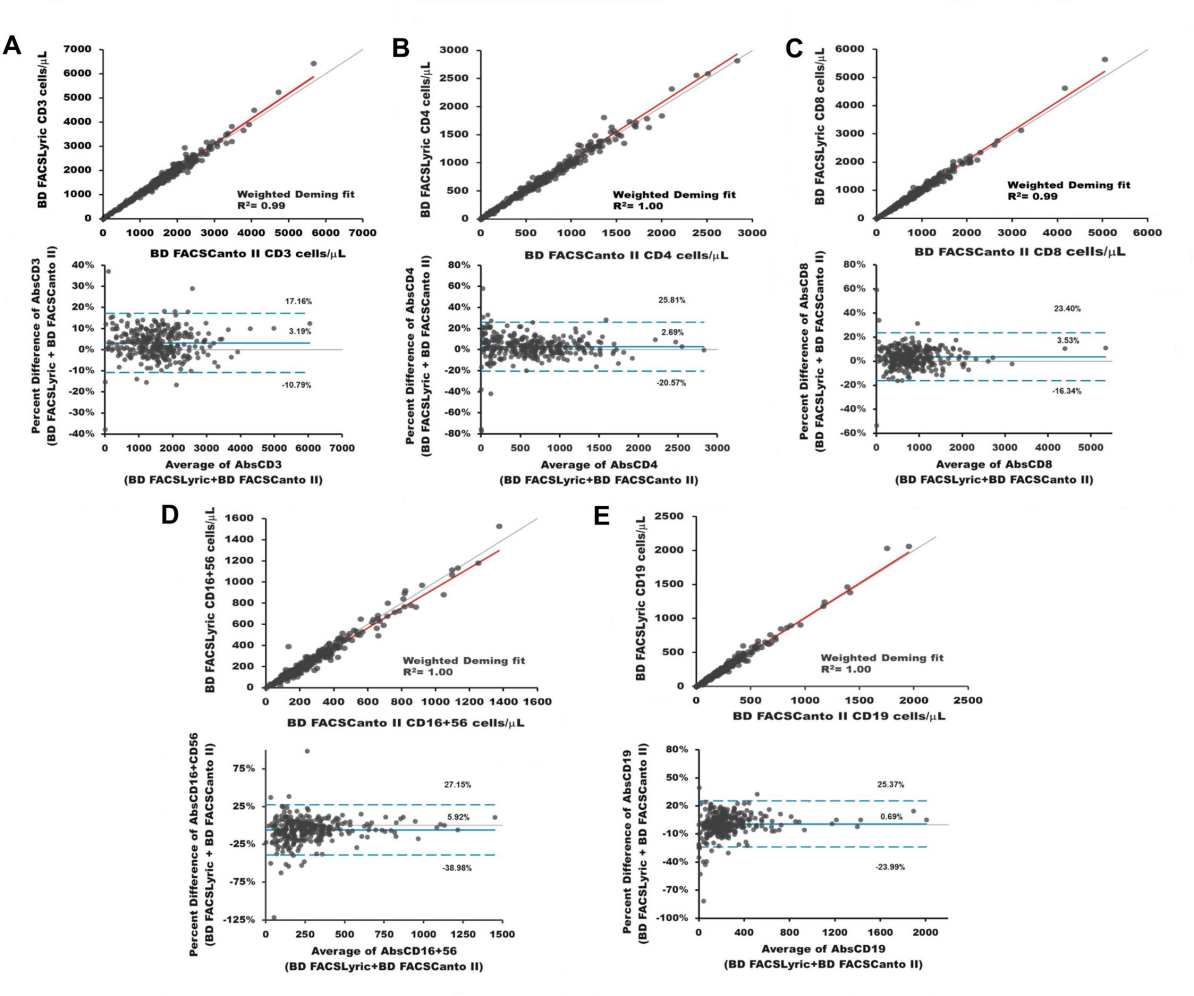
Deming regression and Bland-Altman plots of the absolute counts of T-, B-, and NK lymphocytes identified with the BD Multitest 6-Color TBNK reagent. This is the Fig 2 legend. The T- cells (CD3^+^, CD3^+^CD4^+^ and CD3^+^CD8^+^) shown as A, B, and C; the NK lymphocytes in D, and the B-lymphocytes are depicted in E stained with BD Multitest 6-Color TBNK reagent. The Deming regression are the upper plots with identity line (grey) and the Weighted Deming regression line (red) of each subset. The x-axis depicts BD FACSCanto II cell counts and the y-axis shows the BD FACSLyric counts. The Bland-Altman graphs are the lower plots of each subset, with the bias line (blue solid line) and the upper and lower limits of agreement (blue dotted lines). For these plots, the x-axis corresponds to the average of the lymphocyte marker, and the y-axis represents the estimated percent difference of the absolute counts between systems.

For the method comparison, 356 specimens were enrolled for the BD Multitest 6-color TBNK reagent and 398 for the BD Multitest IMK kit. There were valid results for 362 IMK, and 317 TBNK stained samples grouped in four CD4 cell count bins, from HIV-infected (IMK = 256/TBNK = 226), immune reconstitution (IMK = 19/TBNK = 16), normal (IMK = 46/TBNK = 47) or unknown (IMK = 40/TBNK = 29) subjects. 36 IMK and 39 TBNK samples were excluded from analysis because of instrument problems or not processed as per protocol (poor sample quality, process controls were not tested prior to sample testing, lymphosum failure, samples were processed outside the time window for sample staining and/or acquisition, acquisition did not satisfy the minimum number required of lymph events, beads, time and reagent storage issues). The Deming regression results for the BD Multitest 6-color TBNK reagent gave R^2^≥0.98, and slope values between 0.90 and 1.05. The Deming regression results for the BD Multitest IMK kit were calculated for CD3 average, CD3-tube-1, CD3-tube-2, CD4, CD8, CD16^+^CD56 and CD19. The Deming regression results for the BD Multitest IMK kit gave R^2^≥0.98; with slope values between 0.94 and 1.05 (Table 1). The T-, B-, and NK lymphocyte Deming regression results show a linear correlation between the BD FACSLyric and the standard-of-care system. Fig 2 illustrates the linear relationship by Deming regression of the absolute and percentage of T- (A, B, and C), B- (E), and NK (D) cells stained with BD Multitest 6-color TBNK (tupper plots) and the Bland-Altman results (lower plots) with limits of agreement of the absolute cell counts.

**Table 1:** T-, B-, and NK cell Deming regression results determined using BD Multitest reagents.

Lymphocytes		IMK Kit			6-color TBNK Reagent		
		R^2^	Slope [95% CI^c^]	Intercept	R^2^	Slope [95% CI^c^]	Intercept
**Absolute counts** **(cells/**μ**L)**^a^	**CD3 Ave**	0.99	1.04 [1.03, 1.05]	1.04			
	**CD3 T1**	0.99	1.03 [1.02, 1.05]	3.18	0.99	1.04 [1.03, 1.04]	-0.97
	**CD3 T2**	0.99	1.04 [1.03, 1.05]	-0.62			
	**CD4**	0.99	1.02 [1.01, 1.04]	-0.05	1.00	1.04 [1.02, 1.05]	-0.79
	**CD8**	0.99	1.02 [1.00, 1.04]	-1.35	0.99	1.03 [1.02, 1.05]	0.23
	**CD16**^**+**^**56**	0.99	0.96 [0.94, 0.98]	-3.79	1.00	0.94 [0.90, 0.99]	-0.23
	**CD19**	1.00	1.02 [1.01, 1.04]	-0.05	1.00	1.01 [1.00, 1.03]	-0.21
**Percentage of lymphocytes (%)**^b^	**CD3 Ave**	0.99	1.00 [0.99, 1.01]	0.60			
	**CD3 T1**	0.99	1.00 [0.99, 1.01]	0.68	0.99	1.00 [0.98, 1.01]	1.43
	**CD3 T2**	0.99	1.00 [0.99, 1.01]	0.21			
	**CD4**	1.00	1.01 [1.00, 1.02]	-.0.22	1.00	0.99 [0.99, 1.02]	0.34
	**CD8**	0.99	1.00 [0.98, 1.01 ]	-0.08	1.00	1.01 [1.00, 1.02]	0.09
	**CD16**^**+**^**56**	0.99	0.99 [0.98, 1.01]	-0.81	0.99	1.00 [0.97, 1.03]	-0.73
	**CD19**	1.00	1.02 [1.01, 1.03]	1.02	1.00	1.02 [1.01, 1.04]	-0.29

^a^Weighted Deming regression

^b^regular Deming regression

^c^95%confidence interval.

The results of the Deming regression results from the BD Multitest IMK and BD Multitest 6-color TBNK per lymphocyte subset stained with the BD Multitest reagents are summarized in Table 1 presented as R^2^, slope with the 95% confidence interval and the intercept.

In addition, the concordance or method agreement analysis between the standard-of-care and BD FACSLyric systems around the CD4 cell count clinically relevant cutoff of 200 cells/μL was estimated for both the BD Multitest IMK kit and the 6-color TBNK reagent. The results show the overall percent agreement values of 99% with lower and upper confidence limits of 97% and 100%. Detailed results including positive and negative agreement are depicted in Table 2 for each BD Multitest reagent. The diagnostic effectiveness is presented as percentage of the overall, positive and negative agreements with 95% confidence limits (CL) between the BD FACSLyric and the BD FACSCanto II systems.

**Table 2:** Agreement at the CD4 clinical cutoff of 200 cells/μL.

Reagent	Systems	FACSCanto II (Cells/μL)			Agreement	Percent (%)	95% CL (LCL^d^, UCL^e^)
	BD FACSLyric (Cells/μL)	Positive (≤200)	Negative (>200)	Total			
**IMK**	Positive (≤200)	85	2	87	**Overall**	98.9	97.2, 99.6
	Negative (>200)	2	273	275	**Positive**	97.7	92.0, 99.4
	Total	87	275	362	**Negative**	99.3	97.4, 99.8
**6-Color TBNK**	Positive (≤200)	71	0	71	**Overall**	99.1	97.3, 99.7
	Negative (>200)	3	243	246	**Positive**	96.0	88.8, 98.6
	Total	74	243	317	**Negative**	100	98.4, 100.0

^**d**^lower confidence limit

^e^upper confidence limit.

The predicted percent bias (%bias) forecasts the validity of the CD4 T-cell estimated values at the clinically relevant cutoff of 200 cell counts/μL for each BD Multitest reagent is shown as percent bias (%bias) with the standard error (SE) and the 95% confidence interval (Table 3).

**Table 3:** Predicted bias interval at the CD4 clinical cutoff.

BD Multitest assay	CD4 cutoff (cells/μL)	%Bias (%)	SE	95% CI^c^
**IMK kit**	200	2.55	0.49	1.59, 3.51
**6-Color TBNK reagent**		3.39	0.59	2.23, 4.54

^c^confidence interval.

The interlaboratory data of the T-, B and NK lymphocytes stained with the BD Multitest 6-color TBNK reagent was evaluated as total precision presented as the mean and the percent coefficient of variation (%CV) of the absolute counts, and total precision mean and standard deviation (SD) for the percentage of lymphocytes subsets. The %CV of total precision for the absolute cell counts had values <10%; and for percentage of lymphocytes the SD values were <2.0% (Table 4).

**Table 4:** Inter-laboratory total precision lymphocyte subset results with BD Multitest 6-color TBNK reagent.

Lymphocyte Subset		CD4 Low		CD4 Normal	
		Mean	%CV^f^	Mean	%CV^f^
**Absolute counts** **(cells/μL)**	**AbsCD3**	875.81	4.86	1742.39	5.48
	**AbsCD4**	185.79	7.28	1175.19	5.95
	**AbsCD8**	616.88	5.14	557.86	8.32
	**AbsCD16**^**+**^**CD56**	294.49	6.82	231.03	9.48
	**AbsCD19**	335.03	6.37	276.52	7.72
		**Mean**	**SD**^g^	**Mean**	**SD**^g^
**Percentage of lymphocytes (%)**	**%CD3**	57.46	1.14	76.98	1.02
	**%CD4**	12.19	0.73	51.91	1.09
	**%CD8**	40.47	1.05	24.64	1.59
	**%CD16**^**+**^**CD56**	19.31	0.84	10.20	0.75
	**%CD19**	21.97	0.84	12.21	0.65

^f^Percent coefficient of variation

^g^standard deviation.

**Fig 3:**
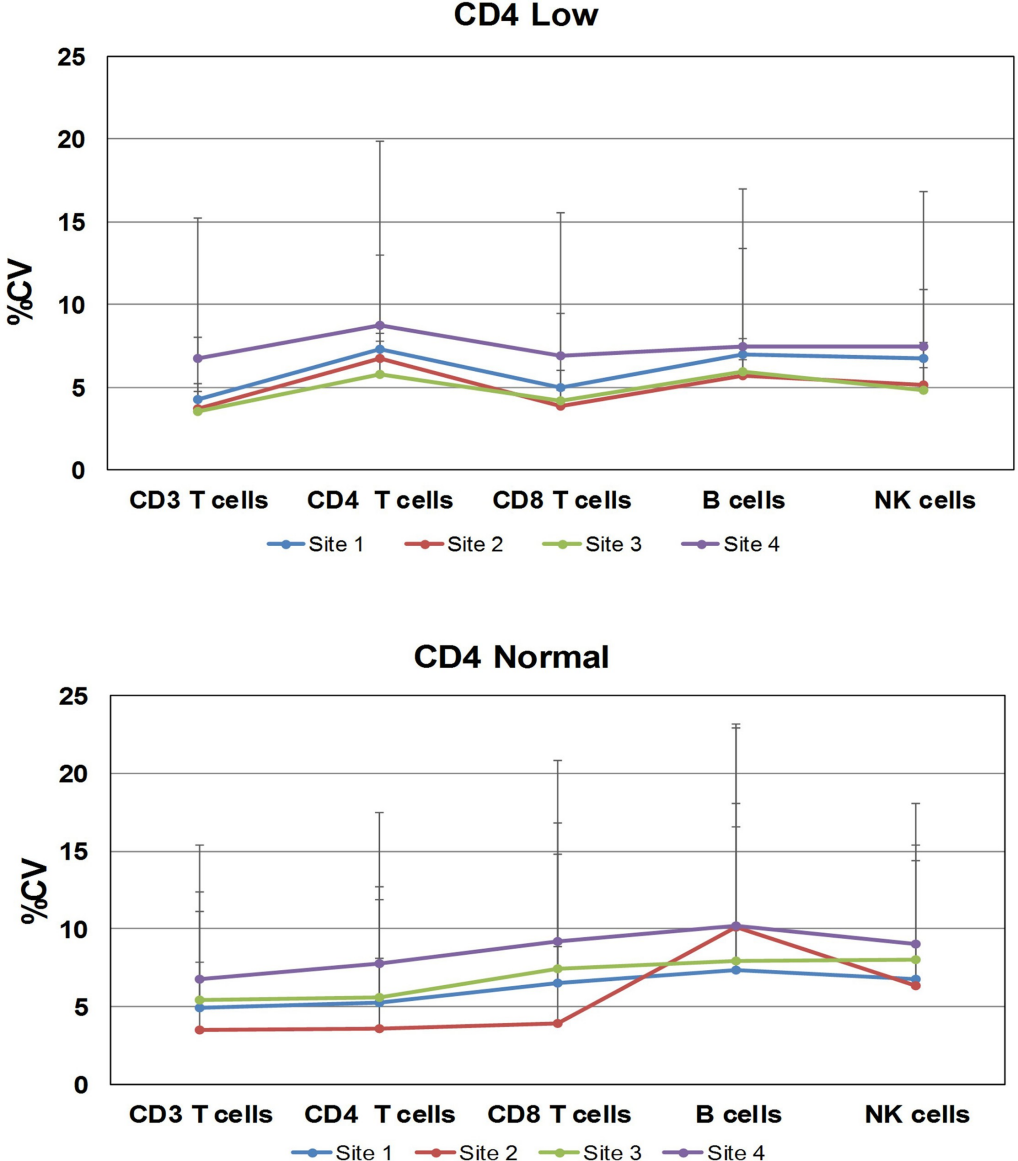
Inter-laboratory total precision per site. The variability per site is illustrated with the BD Multitest 6-color TBNK reagent total precision. The total precision results using CD Chex Plus control materials for the CD4 low are shown on the upper plot, and the total precision for CD4 normal on the bottom plot. The x-axis shows the participating laboratoriers per number and color. The y-axis displays the percentage of coefficient of variance (%CV) and the upper CV limit.

Further analysis of the variability per site is shown as total precision in Fig 3. The CD4 low process control per site showed %CV values between 3.5 and 8.8% for all the lymphocyte subsets. For the CD4 normal process control, the %CV values were between 3.5 and 10.2%, with higher values for NK cells. Interestingly, three sites designated a single operator for the duration of the study, whereas at one site (site 4), three operators prepared the samples. The results from the latter site showed a broader range of the total precision values.

### Stability of storage and reference intervals

The effects of specimen storage on the T-, B-, and NK lymphocytes was evaluated in 186 specimens stained with the BD Multitest IMK kit at the reference time point (T0). 185 specimens were evaluated at time point T1, 178 at time point T2; and 182 at time point T3 using bias analysis. The absolute counts of the T-CD3, CD4, CD8 and NK- cells at time point T1 showed %bias values between -1.01 to 2.7. For CD19, the %bias was -6.54. At the time points T2 and T3, the %bias values were discretely negative between -4.11 and -2.29 for the absolute cell count; and between -0.93 and 1.11 for the percentage of lymphocytes showing minimal changes overtime. The results of this study were consistent for storing the EDTA anti-coagulated blood up to 48 h, and for the stability of the stained sample for up to 24 h at room temperature when using the IMK kit reagents.

In similar fashion, 185 specimens were analyzed at the reference (T0), T1 and T2 time points using the BD Multitest 6-color TBNK reagent. The CD3, CD4, CD8 and NK- absolute counts showed mean percent biases (%bias) values between -0.55 and 1.05. For CD19, the %bias for absolute counts was -5.9. Similarly for the percentage of CD19 cells, the %bias values were between -0.66 and 1.69, showing minimal differences between the tested time points. The study results validated the BD Multitest 6-color TBNK results of 24 h for storing the EDTA anti-coagulated blood at room temperature, and for up to 6 h for maintaining the stained sample at room temperature. Results are depicted in Table 5.

**Table 5:** Specimen and stained sample storage biases at different time points using BD Multitest reagents.

Time point	Cell Type	Mean %Bias	LCL^h^	UCL^i^
**IMK T1** **(N = 185)**	**CD3 Ave**	-0.70	-1.45	0.06
	**CD3 T1**	-0.26	-1.19	0.67
	**CD3 T2**	-1.01	-1.87	-0.15
	**CD4**	-0.31	-1.54	0.92
	**CD8**	0.42	-0.60	1.44
	**CD19**	-6.54	-8.61	-4.47
	**CD16**^**+**^**CD56**	2.70	0.29	5.11
**IMK T2** **(N = 178)**	**CD3 Ave**	-3.46	-4.27	-2.66
	**CD3 T1**	-3.51	-4.44	-2.57
	**CD3 T2**	-3.31	-4.28	-2.35
	**CD4**	-2.94	-4.19	-1.69
	**CD8**	-2.75	-3.73	-1.76
	**CD19**	-11.15	-13.42	-8.87
	**CD16**^**+**^**CD56**	-2.29	-4.66	0.08
**IMK T3** **(N = 182)**	**CD3 Ave**	-4.10	-4.96	-3.24
	**CD3 T1**	-4.11	-5.09	-3.12
	**CD3 T2**	-4.00	-5.03	-2.97
	**CD4**	-2.62	-4.54	-0.70
	**CD8**	-3.28	-4.47	-2.10
	**CD19**	-10.02	-12.94	-7.11
	**CD16**^**+**^**CD56**	-0.48	-7.52	6.57
**6-color TBNK T1** **(N = 180)**	**CD3**	0.02	-0.95	0.99
	**CD4**	-0.55	-1.92	0.81
	**CD8**	0.59	-0.49	1.66
	**CD19**	-5.39	-7.51	-3.28
	**CD16**^**+**^**CD56**	-0.31	-2.85	2.23
**6-color TBNK T2** **(N = 178)**	**CD3**	0.36	-0.71	1.43
	**CD4**	-0.33	-1.66	0.99
	**CD8**	1.05	-0.13	2.23
	**CD19**	-5.91	-8.05	-3.77
	**CD16**^**+**^**CD56**	0.62	-1.81	3.06

^h^lower confidence limit

^i^upper confidence limit.

For reference intervals, a total of 134 adult subjects were enrolled, 69 females and 65 males (19 to 80 years old). The overall T, B-, and NK cell reference intervals were estimated by pooling the male and female results of the absolute cell counts and % of lymphocytes for each lymphocyte subset, which are depicted for each BD Multitest reagent (Table 6). Results are presented for the 90% confidence intervals for the 2.5 and 97.5 percentiles of the absolute counts (Abs) and percentage (%) of the lymphocyte subsets per BD Multitest reagent.

**Table 6:** Lymphocyte subset reference intervals for the BD Multitest reagents.

Reagent and T-, B-, and NK Lymphocytes		Reference Interval		90% CI^j^: Percentile 2.5		90% CI^j^: Percentile 97.5	
		Percentile 2.5	Percentile 97.5	Lower CI^k^	Upper CI^k^	Lower CI^k^	Upper CI^k^
**IMK** **N = 130**	**AbsCD3 Ave**	827	2547	613	875	2513	3357
	**AbsCD3 T1**	840	2641	620	860	2413	3353
	**AbsCD3 T2**	812	2655	606	858	2412	3361
	**AbsCD4**	488	1711	407	549	1588	1835
	**AbsCD8**	154	1097	115	185	981	2141
	**AbsCD16**^**+**^**CD56**	102	617	81	106	603	1316
	**AbsCD19**	60	551	21	108	509	600
	**%CD3 Ave**	56.86	82.5	52.43	58.02	82.13	89.44
	**%CD3 T1**	56.65	83.36	53.28	57.96	81.86	89.96
	**%CD3 T2**	56.74	82.54	51.58	58.07	81.64	88.93
	**%CD4**	32.42	63.19	26.95	32.8	61.16	65.66
	**%CD8**	8.99	38.99	6.82	11.94	37.11	52.31
	**%CD16**^**+**^**CD56**	5.42	29.65	3.92	5.63	24.95	35.96
	**%CD19**	5.14	22.96	1.24	6.79	21.6	24.57
**TBNK** **N = 134**	**AbsCD3**	856	2669	607	894	2342	3090
	**AbsCD4**	491	1734	363	543	1636	1834
	**AbsCD8**	162	1074	111	178	951	1946
	**AbsCD16**^**+**^**CD56**	108	680	83	117	583	1191
	**AbsCD19**	73	562	32	102	536	757
	**%CD3**	57.52	83.11	51.31	58.9	82.42	87.15
	**%CD4**	31.45	62.38	26.4	33.23	61.4	66.86
	**%CD8**	9.55	38.32	6.65	11.27	36.33	51.7
	**%CD16**^**+**^**CD56**	5.17	30.36	4.51	5.82	27.16	35.08
	**%CD19**	5.89	24.21	2.24	6.92	22.73	29.45

^**j**^ **90%** confidence interval

^**k**^confidence interval.

The analysis by gender of reference intervals showed that females have higher T-cell absolute counts for CD3 (1684–1691) and CD4 (1112–1115), and higher percentage values for %CD3 (73–74%), %CD4 (48–49%), than the males, which have T-cell absolute counts of CD3 (1413–1430), CD4 (886–892) and percentage values for %CD3 (69–70%) and %CD4 (44%). The differences were statistically significant (p<0.01) for the two reagents. The opposite was observed for the %NK values; the female percentage (<12%) was lower than male values (<15.5%). No-gender differences were found for the CD8 T-cell and B-cell counts or percentage values. The analysis by age did not identify statistically significant differences (S1 Table).

## Discussion

Phenotyping of lymphocytes using flow cytometry is extensively used [5, 8, 19, 20] for enumeration of the CD4 absolute cell counts to determine HIV-infection or AIDS disease status, for monitoring disease progression or co-infections, for patient staging, and for initiation of anti-retroviral treatment (ART). In 2014, the WHO and NIH issued guidelines recommending initiation of the ART regardless of the CD4 cell counts and a shift to use viral load for HIV+ subject diagnostic and monitoring [9, 10, 21]. There has been substantial progress in expanding access to care and ART. However, the HIV-infected subjects have higher risk of co-morbidity and are more vulnerable to opportunistic infections [9, 22]. In addition, there is slow adoption of viral load as a routine central laboratory test for patient staging prioritization when ART availability is limited [8, 22]; therefore, enumeration of the CD4 cell counts still has a role when viral load is not easily available. Immune deficiencies encompass a broad spectrum of conditions, so T-, B- and NK cells can be measured for screening of immune status, and if compromised, followed by more advanced flow cytometry analyses [6, 7]. Flow cytometric enumeration of lymphocyte subsets is also useful following administration of B-,cell depletion agents [23], and for monitoring immune cell reconstitution after transplant [24, 25].

The BD Multitest IMK kit and 6-color TBNK reagents enumerate the T-, B-, and NK cells in a two- or one-tube format and provide a snapshot of the status of the patient’s immune system. Our method comparison results showed that the BD FACSLyric and BD FACSCanto II systems generate equivalent results (Tables 1 and 2), with acceptable inter-laboratory variability (%CV <10%; Table 4). Also, the inter-laboratory results by site offered insights on the challenge of the intrinsic variability when multiple operators are involved in manually preparing the samples for testing (Fig 3). One limitation was the limited and uneven number of participating operators per site, preventing between-operator analysis. The storage of whole blood and stained specimens at room temperature also pose challenges. Our results verified the stability of the specimens anti-coagulated with EDTA and maintained at the laboratory room temperature for up to 48 hours for the IMK reagent, and up to 24 hours for the TBNK reagent. The stability of the stained sample verified a time window up to 6 h after staining blood stored for 48 h or 24 h when stained with IMK kit or 6-color TBNK reagent, respectively. The reference intervals for the BD Multitest reagents showed gender results that are statistically significant different for CD3 and CD4 T-cell counts and %CD4 cells. These results are similar to previously published reports in different countries [26–28]. However, only the CD4 T-cell count is clinically relevant for HIV-infected (CD4 200 cells/μL), and historically the CD4 T-cell count gender differences have not been considered clinically relevant to drive clinical decisions for initiation of care [20]. Furthermore, the current WHO or NIH guidelines recommend the initiation of ART regardless of the CD4 counts [9, 10, 21]. The role of CD4 enumeration is still important where there is limited availability of ART for staging of drug treatment and monitoring, and for monitoring opportunistic infections in the HIV infected subjects at risk (CD4 <200 cells/μL) [8–10]. One limitation of this study is the small number of specimens from subjects being tested for immune reconstitution, leucopenia or leukocytosis.

The BD FACSLyric system offers some technical advantages including the automated universal assay setup feature using the BD CS&T and BD FC Beads. The initial setup of the BD FACSLyric cytometer consists of running FC Beads which measures the optical spillover of the dyes used in the assays. The system uses the measured spillover values to mathematically remove the cross channel spillover when running assays. This ensures optimal separation of fluorescent populations and eliminates the need to run daily compensation controls. FC Beads are re-run every 60 days to ensure the measured spillover values are kept up to date. Once initial setup is compete, users run a daily single-step procedure that takes approximately five minutes using BD CS&T beads and BD FACSuite Clinical software.

The setup procedure automatically quantifies and tracks cytometer performance and to measure and adjust the cytometer photomultiplier tube voltages (PMTVs), thereby ensuring that the target values of the median fluorescence intensity (MFI) are maintained over time. If the MFI target values are exported to additional BD FACSLyric cytometer(s) within the same clinical laboratory or across laboratories, the BD CS&T beads are capable of adjusting the PMTVs of each new instrument automatically to maintain the target values. The target MFI’s for each channel used in the clincal assays were determined based on fluorochromes intensity and level of optical spillover into and out of adjacent detector channels. During the clinical evaluation, the operators at each participating laboratory defined the BD FACSLyric cytometer baseline performance using the BD CS&T beads and performed daily QC. BD FC Beads were used periodically once a month for SOV for the duration of the clinical evaluation. These features reduced instrument-to-instrument variability, by ensureing that all systems used in the study were placing cell populations at the same target MFIs. These features enable standardization of the instruments and for assay portability across laboratories. In the context of this multi-site evaluation utilizing the BD Multitest reagents, the preceding elements describing the universal assay setup and its advantages lend credence that differences in results are consistent with the specimen biology, rather than changes in the instrument performance.

In summary, the method comparison showed equivalent performance between the BD FACSLyric system and the standard-of-care, the BD FACSCanto II system, for enumeration of the T-, B and NK cells in human blood anti-coagulated with EDTA from normal subjects, and from HIV-infected, immune reconstitution, leukocytosis or leucopenia patients using the BD Multitest reagents. Interlaboratory total precision results across the four study sites were satisfactory. The blood storage results were consistent with the established claims for the stability of the BD Multitest reagents (up to 48 h for IMK and 24 h for TBNK), and also confirmed the time window for the stability of the stained sample at 24 h or 6 h, depending on the time the specimen had been stored. Lastly, the reference intervals were determined in healthy adult males and females, free of hematological abnormalities of the lymphocyte subpopulations.

The BD FACSLyric system CE marked in compliance with the European Directive 98/79/EC (Directive of the European parliament and of the Council of 27 October 1998 on in vitro diagnostic medical devices). The BD FACSLyric flow cytometer is for In Vitro Diagnostic Use with BD FACSuite Clinical software for up to 6 colors.The BD FACSLyric flow cytometer is for Research Use Only with BD FACSuite software for up to 12 colors.

CD-Chex Plus is a registered trademark of Streck, Inc.

Cy is a trademark of GE Healthcare.

“BD, FACSLyric and all other trademarks are property of Becton, Dickinson and Company. 2018 BD and its subsidiaries. All rights reserved.”

## Supporting information

S1 File Method comparison and intersite reproducibility study protocol. (PDF)

S2 File Stability of blood and stained sample storage study protocol. (PDF)

S3 File Reference intervals study protocol. (PDF)

S4 File Method comparison dataset. (XLSX)

S5 File Interlaboratory variability dataset. (XLSX)

S6 File Stability of blood and stained sample storage dataset. (XLSX)

S7 File Reference intervals dataset. (XLSX)

S8 File STARD FACSLyric -2015 checklist. (DOCX)

S1 Table Reference intervals by gender. (DOCX)
