# Supplementary material for: Accurate and reproducible enumeration of T-, B-, and NK lymphocytes using the BD FACSLyric 10-color system: A multisite clinical evaluation
Source: PLoS One. 2019 Jan 28;14(1):e0211207. doi: 10.1371/journal.pone.0211207 (PMC6349332; doi:10.1371/journal.pone.0211207)
Supplement: S1 File — (PDF) [file pone.0211207.s001.pdf]

Corporate Clinical Development
CCD-5115.T01 Protocol Template
Revision 04

BD Protocol #: **CAS-LIBHIVACC**

Protocol Title: **Method Comparison of the BD Multitest reagents on the Investigational BD FACSLyric Clinical Flow Cytometer.**

**Current Version**	Version 2.0/19Aug2016 (Amendment)
**Version History**	Version 2.0/19Aug2016 (Amendment)<br>Version 1.1/18Apr2016 (Administrative Change)<br>Version 1.0/21Sep2015 (Original)
**Sponsor**	BD – Biosciences<br>2350 Qume Drive<br>San Jose, CA 95131
**Sponsor Medical Monitor**	VP, Medical Affairs<br>2350 Qume Dr.<br>San Jose, CA 9513<br>201-847-4454
**Sponsor Risk Assessment**	☐ Significant Risk (SR) ☐ Non-significant Risk (NSR)<br>☒ Minimal Risk

The product information and data disclosed through this protocol are confidential and may not be disclosed without prior written consent of Becton, Dickinson and Company.

This study will be performed in accordance with all stipulations of the protocol and in compliance with all applicable BD Policies and Procedures. This study will be conducted in accordance with the ethical principles that originate from the Declaration of Helsinki and the Belmont Report. Study conduct will comply with US FDA Regulations, EU Directive 98/79/EC, applicable state and local regulations, and the Good Clinical Practice guidelines set forth by the International Conference on Harmonization (ICH-E6).

The performance characteristics of this product have not been established. Results must not be used as a diagnostic procedure without confirmation of the diagnosis by another, medically established diagnostic product or procedure.

All Study Product(s) used in this study should be considered to be for investigational use only.

SPONSOR PROTOCOL APPROVAL

Signature below indicates approval of the protocol as written.			
**Individual or function**	**Name**	**Signature**	**Date**
Medical Affairs Team Representative		*This document is signed electronically in the eTMF system*	
Study Statistician		*This document is signed electronically in the eTMF system*	
Regulatory		*This document is signed electronically in the eTMF system*	
Study Manager		*This document is signed electronically in the eTMF system*	
Business Unit Medical Director and Medical Monitor		*This document is signed electronically in the eTMF system*	

INVESTIGATOR SIGNATURE PAGE

**Principal Investigator**	{Name}<br>{Address} (if different from site)
**Investigational Site**	{Site Name}<br><br>{Address}

I have read this protocol and agree to conduct this study in accordance with all stipulations of the protocol and in compliance with all applicable Good Clinical Practices and regulations.

____ ______________

Signature of Principal Investigator Date

Table of Contents

LIST OF ABBREVIATIONS AND DEFINITION OF TERMS 7

1.0 INTRODUCTION 8
- 1.1 Statement of Compliance 8
- 1.2 Proposed Intended Use 8
- 1.3 Explanation of Sub-Studies 8
- 1.4 Investigational Device System and Description 8
- 1.5 Predicate Device System 9

2.0 OBJECTIVES 9
- 2.1 Primary Objectives 9
- 2.2 Secondary Objectives 10

3.0 STUDY DESIGN 10
- 3.1 Overall Study Design 10
- 3.2 Specification of Study Endpoints 10
  - 3.2.1 Primary Endpoints 10
  - 3.2.2 Secondary Endpoints 11
  - 3.2.3 Safety Endpoints 11
- 3.3 Treatment Allocation and Methods to Reduce Bias 11
  - 3.3.1 Randomization 11
  - 3.3.2 Skill and Behavior of Persons Interacting with the Device (if applicable) 11
- 3.4 Stopping Rules 11

4.0 STUDY POPULATION 11
- 4.1 Inclusion Criteria 12
- 4.2 Exclusion Criteria 12

5.0 DESCRIPTION OF STUDY PRODUCTS 13
- 5.1 Test Product(s) 13
- 5.2 Reference Products (or Methods) 13
- 5.3 Ancillary Products 14
- 5.4 Product Labeling 14
- 5.5 Maintenance and Storage of Study Products 14

6.0 STUDY METHODS 15
- 6.1 Summary of Daily Study Activities 15
- 6.2 Specimen Enrollment 15
- 6.3 Instrument Setup and Process Control 15
- 6.4 Inter-Laboratory Reproducibility: Sample Staining and Acquisition 17
- 6.5 Sample Staining and Acquisition 18
- 6.6 Data Review and Analysis 19
- 6.7 Required Training and Proficiency Testing 20
- 6.8 Customer Ease of Use/Usability 20
- 6.9 Additional Site Responsibilities 20
  - 6.9.1 IUO BD FACSLyric Monthly Maintenance 20
  - 6.9.2 Predicate Instrument Maintenance 20

6.9.3 Communication with BD 20

7.0 INTERRUPTION OR DISCONTINUATION OF PARTICIPATION/TESTING 21
7.1 Discontinuation of Specimen Testing 21
7.1.1 Replacement of Discontinued Subjects/Specimens 21
7.1.2 Retention of Data from Discontinued Subjects/Specimens 21

8.0 RISK / BENEFIT ASSESSMENT 21
8.1 Potential Risks 21
8.2 Potential Benefits 21

9.0 ASSESSMENT OF SAFETY AND ADVERSE EVENTS 21

10.0 INCIDENTS 22

11.0 RETURN OR DESTRUCTION OF STUDY PRODUCT 22

12.0 DATA COLLECTION AND MANAGEMENT 22
12.1 Source Documents 22
12.2 Case Report Forms (CRF) 23
12.3 Electronic Source Data (optional) 23
12.4 Data Management and Storage 23

13.0 STATISTICAL METHODS 24
13.1 Sample Size Determination 24
13.2 Data Evaluability 24
13.3 Statistical Methods 24
Additional analysis may be carried out for information only as depicted in the Statistical Plan. ..25
13.4 Demographics/Other descriptive information 25
13.5 Interim analysis 25

14.0 QUALITY CONTROL AND ASSURANCE 25
14.1 Accountability of Study Products 25
14.2 Monitoring 25
14.3 Audits and Inspections 26
14.4 Protocol Deviations 26

15.0 ETHICAL AND REGULATORY STANDARDS 26
15.1 IRB/EC 26
15.2 Informed Consent 26
15.3 Confidentiality of Data 27
15.4 Protocol Modifications 27
15.5 Study Discontinuation 28
15.6 Clinical Study Registration 28
15.7 Publication of Results 28
15.8 Record Retention 28

16.0 BIBLIOGRAPHY/REFERENCES 29

17.0 PROTOCOL REVISION HISTORY 29

18.0 APPENDICES 29

18.1 Study Design 30
18.2 Instruments, controls, and reagents used, broken down by Sub-Study and step of study .31

LIST OF ABBREVIATIONS AND DEFINITION OF TERMS

AE	Adverse event
BD	Becton Dickinson and Company
CFR	Code of Federal Regulations
CI	Confidence Interval
CRF	Case Report/Record Form
CRO	Contract Research Organization
CS&T	Cytometer Setup and Tracking
DCF	Data Clarification Form
EDC	Electronic Data Capture
EDTA	Ethylene diamine tetra acetic acid
FDA	Food and Drug Administration
FDAAA	FDA Amendments Act of 2007
FTP	File Transfer Protocol
GCP	Good Clinical Practice
HIPAA	Health Insurance Portability and Accountability Act
ICH	International Conference on Harmonization
IFU	Instructions for Use
ILR	Inter-Laboratory Reproducibility
IRB/EC	Institutional or Independent Review Board/Ethics Committee
IUO	Investigational Use Only
IVD	In Vitro Diagnostics
IVDD	In Vitro Diagnostic Directive
µl	microliter
mL	Milliliter
mm	Millimeter
PEO	Performance Evaluation Only
PHI	Protected Health Information
PI	Package Insert
QNS	Quantity Not Sufficient
SAE	Serious Adverse Event
SD	Standard deviation
SOP	Standard Operating Procedure
WFN	Work Flow Notes
WHO	World Health Organization

1.0 INTRODUCTION

1.1 Statement of Compliance

This study is to be conducted in compliance with this protocol, Good Clinical Practices (GCP)[1], the Declaration of Helsinki, Title 21 of the Code of Federal Regulations §§ 50, 56, and 812, and the International Conference on Harmonization E6 and ISO Standards 14155-1:2003 and 14155-2:2003.

1.2 Proposed Intended Use

The BD FACSLyric flow cytometers (3-1, 4-2, 4-2-2 and 4-3-3 configurations) function as part of a system with BD FACSuite Clinical software intended for use with cleared or approved in vitro diagnostic (IVD) assays that are indicated for use with the instrument for identification and enumeration of human cell subsets. Only six fluorescence detection channels and two scatter channels using a blue (488 nm) and a red (633 nm) laser are cleared for in vitro diagnostic use.

1.3 Explanation of Sub-Studies

This protocol targets high volume clinical sites that currently use the IVD BD Multitest IMK kit, BD Multitest 6-color TBNK, and BD reagents, use the IVD FACSCalibur and/or BD FACSCanto II, and have access to remnant samples from patients exhibiting leucopenia or leukocytosis, or subjects free of hematological abnormalities (normal patients/donors), or HIV patients or patients undergoing immune reconstitution.

The method comparison of the IVD BD Multitest IMK and IVD BD Multitest 6-color TBNK assays when comparing the results between the investigational and predicate systems. The BD Multitest IMK kit includes Reagent A) Multitest CD3/CD8/CD45/CD4 and Reagent B) Multitest CD3/CD16+56/CD45/CD19.

The investigational system will be the IUO BD FACSLyric flow cytometer/IUO Universal Loader/ IUO FACSuite Clinical software/IUO BD CS&T Beads/IUO BD FC Beads.

The predicate system will be the FACSCanto II flow cytometer/ FACSLoader (optional)/ FACSCanto Clinical software/FACS 7-Color Setup Beads.

In addition, the protocol integrates an assessment of inter-laboratory reproducibility and an assessment of the customer's experience with the IUO BD FACSLyric system (usability).

In instances when information pertains to only one of the Sub-Studies, the information will be presented in parallel columns, as demonstrated above.

A table in the Appendix summarizes the study design of both Sub-Studies (section 18.1). A table in section 18.2 lists the instruments, controls, and reagents used in each Sub-Study.

1.4 Investigational Device System and Description

The investigational device system will be labeled for "Investigational Use Only (IUO)"/for "Performance Evaluation Only" (PEO) to satisfy FDA and IVDD requirements for products that are not cleared/approved. In this document, "IUO" will be used across the document to indicate the test product(s). The IUO system will consist of the following:

Instrument related:

- IUO BD FACSLyric 10 Color flow cytometer with IUO Universal Sample Loader

- Computer workstation with IUO FACSuite Clinical software.

IUO set-up beads:

- IUO BD CS&T beads
- IUO BD FC beads (7-Color Kit for BD FACSuite)

The IUO BD FACSLyric flow cytometer has four configurations (3-1, 4-2, 4-2-2 and 4-3-3). The 4-3-3 configuration will be tested in clinical trials. This configuration has three lasers, two light scatter detectors, 10 fluorescence detectors with optical filters.

The IUO FACSuite Clinical software, which runs on a companion workstation, is an integrated suite of applications and utilities that will enable clinical laboratories to easily run automatic setup and clinical assays using the IUO BD FACSLyric flow cytometer.

IUO BD CS&T beads and IUO BD FC (Fluorescence Control) beads are intended for use with IUO BD FACSLyric and IUO FACSuite Clinical software. The IUO BD CS&T beads are used to check cytometer performance and automatically make adjustments. They are run daily for the Performance QC and twice-a-year for characterization QC. The IUO BD FC beads allow the software to determine and automatically update spillover values for fluorescent compensation for clinical assays. They are run monthly.

1.5 Predicate Device System

The predicate device system, IVD controls, and IVD reagents for the indicated Sub-Study consist of the following:

Instrument related:

- IVD BD FACSCanto II with optional sample loader
- Computer workstation with FACSCanto Clinical software v2.4 or later
- IVD BD 7-Color Setup Beads

IVD controls and reagents:

- BD MultiCheck controls (Normal and CD4 Low levels)
- BD Multitest IMK kit
- BD Multitest 6-color TBNK reagent
- BD Trucount tubes
- Custom Lot of BD MultiCheck process controls (Normal and CD4 Low levels) or Streck CD Chex Plus controls (Normal and CD4 Low levels)

2.0 OBJECTIVES

2.1 Primary Objectives

Determine the method comparison between the investigational use only (IUO) BD FACSLyric system and the predicate IVD BD FACSCanto II system on the determination of lymphocyte sub-populations using

IVD BD Multitest 6-color TBNK and BD Multitest IMK assay reagents with Trucount tubes with remnant, de-linked patient specimens at a minimum of three external study sites.

2.2 Secondary Objectives

- Assess inter-laboratory reproducibility at a minimum of three test sites.
- Evaluate the customer's ease of use (usability) of the IUO BD FACSLyric system.

3.0 STUDY DESIGN

3.1 Overall Study Design

This is a multicenter, prospective evaluation of the IUO BD FACSLyric flow cytometer system vs. a predicate flow cytometer system at three or more clinical sites. This protocol contains two Sub-Studies (A and B), and each Sub-Study has a different predicate flow cytometer and different reagents, as shown above. Each Sub-Study requires at least 240 remnant specimens from patients exhibiting leucopenia or leukocytosis, or subjects free of hematological abnormalities (normal patients/donors), or HIV patients or patients undergoing immune reconstitution, in order to satisfy AbsCD4 and %CD4 binning requirements. For testing of Trucount Control reagents, each day of testing requires a remnant specimen from a patient free of hematological abnormalities (normal patient/donor).

Remnant specimens will be de-identified and delinked prior to enrollment into the study. If quantities and time are sufficient, specimens may be enrolled in both Sub-Studies.

For inter-laboratory reproducibility testing, three replicates of each level control material will be tested during two runs per day on the IUO BD FACSLyric for a minimum of five non-consecutive days at three clinical sites, with a minimum of one operator per site and minimum three instruments between all sites.

For usability evaluation, operators that are proficient with the investigational system will provide qualitative evaluation of the ease of use of the IUO BD FACSLyric system.

3.2 Specification of Study Endpoints

3.2.1 Primary Endpoints

For the Multitest reagents, the Method Comparison endpoints are the bias (expected difference) between the investigational system versus the predicate system measured as absolute lymphocytes subset counts and percentage of the lymphocyte sub-population.

Subsets:

Multitest IMK kit reagent: AbsCD3, AbsCD4, AbsCD8, AbsCD16+CD56, AbsCD19, %CD3, %CD4, %CD8, %CD16+CD56, %CD19. AbsCD3 and %CD3 will be determined by averaging the value obtained from tubes for reagents A and B for the same replicate.

Multitest 6-color TBNK reagent: AbsCD3, AbsCD4, AbsCD8, AbsCD16+CD56, AbsCD19, %CD3, %CD4, %CD8, %CD16+CD56, %CD19.

For each Sub-Study, remnant and de-identified patient specimens will be enrolled to satisfy AbsCD4 and %CD4 binning requirements, as indicated in Section 4.0.

3.2.2 Secondary Endpoints

- Inter-laboratory reproducibility (ILR): Evaluate the closeness of agreement of the IUO BD FACSLyric system measuring AbsCD4 and %CD4 cells using two levels of ILR specimens (either custom lots of BD MultiCheck controls or Streck CD Chex Plus controls) under reproducibility conditions from at least three sites.
- Usability: Determination of the customer's qualitative assessment of how easily and safely an operator could use the IUO BD FACSLyric system.

3.2.3 Safety Endpoints

During study testing, occurrence and severity of any adverse events will be evaluated, recorded, and followed up as required by BD procedures (Corporate Clinical Development and BD Biosciences).

3.3 Treatment Allocation and Methods to Reduce Bias

3.3.1 Randomization

Remnant patient specimens will be de-identified and delinked from patient protected health information and assigned a Study ID number prior to enrollment in the study.

3.3.2 Skill and Behavior of Persons Interacting with the Device (if applicable)

The operators at each site conducting the study should be qualified and have experience with flow cytometric testing, including identifying appropriate specimens, staining samples, acquire data on a flow cytometer, and analyzing flow cytometry results.

3.4 Stopping Rules

No stopping rules for the study have been developed by the Sponsor. The Principal Investigator is responsible for suspending study enrollment for reasons of subject/clinician safety and well-being.

4.0 STUDY POPULATION

The study will be conducted at high volume reference laboratories specializing in flow cytometric immunophenotyping. Study sites will obtain remnant specimens from patients exhibiting leucopenia or leukocytosis, or subjects free of hematological abnormalities (normal patients/donors), or HIV patients or patients undergoing immune reconstitution.

Specimens must be obtained with sufficient time to enroll and stain the specimen from time of blood draw. These specimens will be de-linked and enrolled for study testing.

Bins are designed to ensure that the samples will cover the reportable range. For each reagent, evaluable specimens will be enrolled to satisfy AbsCD4 and %CD4 binning requirements, as indicated in the chart below, based on the average value obtained from the two replicates acquired on the predicate instrument. It is expected that each site will enroll a minimum of 60 specimens with evaluable results per assay, for a minimum total of 240 specimens with valid results across at least three clinical sites.

	Bins (cells/μL)	Target	Min
AbsCD4	1) 0 ≤ CD4 < 200	60	50
	2) 200 ≤ CD4 < 500	60	40
	3) 500 ≤ CD4 < 1000	60	40
	4) 1000 ≤ CD4 < 4500	60	40
%CD4	**Bins (%CD4)**	**Target**	**Min**
	1) 0% ≤ CD4% < 20%	80	50
	2) 20% ≤ CD4% < 35%	80	50
	3) 35% ≤ CD4% < 100%	80	50

In order to fill bins, up to 15% of specimens with evaluable results within each bin may be manipulated. Manipulation must follow an approved method.

Unless specified otherwise, these criteria apply at screening and throughout the study.

4.1 Inclusion Criteria

Peripheral whole blood specimens must satisfy all of the following conditions to be considered for participation:

a. Leftover (i.e., residual) and has not been prospectively procured for study enrollment, but has been collected and tested for CD4 counts and any remaining specimen would routinely be discarded

b. Collected in a blood collection tube with EDTA anticoagulant and stored at room temperature (20-25°C) until enrollment

c. Provided with the specimen draw date and time

d. Of acceptable quality for flow cytometry testing (e.g., no hemolysis or clots and acceptable pre-analytical handling)

e. Of sufficient residual volume for the Sub-Studies: approximately 1.5 mL

f. Drawn within an adequate time to perform post-enrollment staining for specific assay
BD Multitest IMK kit: within 48h
BD Multitest 6C-TBNK reagent: within 24h

4.2 Exclusion Criteria

ANY of the following is regarded as criterion for excluding a specimen from this study:

a. Clotted or hemolyzed specimens

b. Frozen or refrigerated samples

c. Fixed samples

Enrolled specimens may be subsequently excluded from study testing if unsuitable for testing, for example visual inspection prior to study acquisition show clotting or hemolysis. See Section 7.0: Discontinuation of Testing for further information.

5.0 DESCRIPTION OF STUDY PRODUCTS

5.1 Test Product(s)

- IUO labeled BD FACSLyric 10 color flow cytometer with 4-3-3 configuration (BD Catalogue # 651164, 651165)
- IUO labeled Universal Loader (BD Catalogue # 651166)
- BD FACSuite workstation
- IUO FACSuite Clinical software
- IUO assay module
- IUO Multitest assay module
- IUO 6C TBNK assay module
- IUO BD CS&T beads (BD Catalogue # 656504/656505)
- IUO BD FC beads (7-Color Kit for BD FACSuite)
- IUO FACSLyric Instructions for Use
- IUO FACSLyric Safety and Limitations Guide
- IUO Assay Setup Report
- IUO QC Report
- IUO Lab Report
- IUO Physician Report
- IUO Supplemental Report

5.2 Reference Products (or Methods)

- BD Multicheck Normal process controls (BD Catalogue # 340911, 340912, 340913)
- BD Multicheck CD4 Low process control (BD Catalogue # 340914, 340915, 340916)
- Custom lot of BD MultiCheck process controls (CD4 Low and Normal)
- Streck CD Chex Plus controls (Normal and CD4 Low levels)
- BD Trucount Tubes (BD Catalogue # 340334)
- BD FACSFlow sheath fluid (BD Catalogue # 342003)
- BD FACSCanto II with (optional) FACS Loader (BD Cat #338960, 342065)
- FACSCanto II workstation with BD FACSCanto Clinical software (v2.4 or later)
- BD FACS 7-Color Setup Beads (BD Catalogue # 335775 or respective equivalent catalogue # by region)
- BD Multitest IMK Kit (BD Catalogue # 340503 or respective equivalent catalogue # by region)
- BD Multitest 6-color TBNK reagent (BD Catalogue # 644611 or respective equivalent catalogue # by region)BD FACS Lysing Solution (BD Catalogue # 349202)

5.3 Ancillary Products

Ancillary products are materials that are critical to the use of the study product or execution of the protocol, such as certain concomitant medications or critical device components which are used with the study device and must be used exactly as specified in this protocol.

BD will provide the following as needed:

- Electronic pipette(s) (BD Catalogue # 646539 or similar)
- External Hard Drives, USB drives, CDs, or DVDs (to store study data)

Site should have access to the following:

- Calibrated pipettes and pipette tips (to dispense 20-450µl)
- Vortex mixer
- Lab timer
- clock
- bleach
- printer and related supplies
- refrigerator with controlled temperature range between 2-8˚C
- Disposable 12x75-mm BD Falcon capped polystyrene 5ml tubes (Catalogue # 352058 or similar)
- Di H_2O
- Internet connection (optional)

5.4 Product Labeling

Investigational products (including marketed products used off-label) and units shall be labeled in accordance with regulatory requirements, including the following statement, "For Investigational Use Only. The performance characteristics of this product have not been established." Or "For Performance Evaluation Only" (PEO).

For investigational products, labeling will also include the following at minimum, when applicable:

- Study Number
- Product Identification (Product name, catalog number, instrument plate, etc.)
- Manufacturer name and location
- Batch/Lot #
- Use by/Expiration Date

Commercial products will be supplied as labeled by the manufacturer.

5.5 Maintenance and Storage of Study Products

The IUO BD FACSLyric, IVD BD FACSCanto II along with associated workstations and software, should be stored at 16-29˚C. Flow cytometers should be up-to-date on regular maintenance.

BD FACSLysing Solution and BD FACSFlow sheath fluid should be stored at 16-29˚C. BD Trucount Tubes should be stored at 2-25˚C. Unless otherwise indicated, all other controls and reagents should be stored at 2-8˚C. No study product shall be used beyond its expiration date.

6.0 STUDY METHODS

6.1 Summary of Daily Study Activities

Each day's work will include completing relevant CRFs during the process.

- Enroll specimens that meet criteria (quantity, time, other inclusion/exclusion criteria), and re-label with Study ID number.
- Start-up instruments and perform instrument setup using appropriate beads. Confirm that instrument passes.
- Stain MultiCheck controls and acquire on each instrument. Analyze data to determine if they are within manufacture's ranges.
- Inter-Laboratory Reproducibility: Stain 3 sets of the ILR specimens, acquire and analyze data. (Perform testing twice per day of testing for at least 5 non-consecutive days.)
- Stain and acquire study samples.
  - BD Multitest 6-color TBNK and BD Multitest IMK stained samples from a study specimen should be acquired together.
- Review and analyze study specimen data. Store data on site and provide to BD.

6.2 Specimen Enrollment

BD Multitest and BD testing requires remnant EDTA specimens with sufficient quantity from patients exhibiting leucopenia or leukocytosis, or subjects free of hematological abnormalities (normal patients/donors), or HIV patients or patients undergoing immune reconstitution,

For all specimens,

- Confirm that specimen meets inclusion and exclusion criteria.
- De-identify specimen and assign a Study ID number. The site will maintain a coded list that associate the specimen identity with the Study ID number. This list will not be transferred to the sponsor.

6.3 Instrument Setup and Process Control

The steps for Instrument Setup are depicted in Figure 1. The steps for Preparation and Acquisition of Process Controls and Trucount Controls are depicted in Figure 2.

IUO BD FACSLyric:

- Start instrument and workstation. Allow instrument to warm up for 20 minutes prior to running samples or controls.
- In FACSuite Clinical software, run Performance QC with BD CS&T beads. Confirm that the instrument passed. Save report.
- Stain MultiCheck process controls (2 levels) with Sub-Study appropriate reagents. Complete CRFs.
- Reagents: BD Multitest 6-color TBNK and BD Multitest IMK kit reagents.
- Acquire data on controls. Review and analyze data to confirm measured values are within manufacturer's ranges.

FACSCanto II:

- Start instrument and workstation. Allow instrument to warm up for 30 minutes prior to running samples or controls.
- In FACSCanto Clinical software, run 7-Color setup beads. Confirm that the instrument passed. Save report.
- Stain MultiCheck process controls (2 levels) with BD Multitest 6-color TBNK and BD Multitest IMK reagents. Complete CRFs.
- Acquire data on controls. Review and analyze data to confirm measured values are within manufacture's ranges.

Figure 1. Instrument Setup and Acquisition

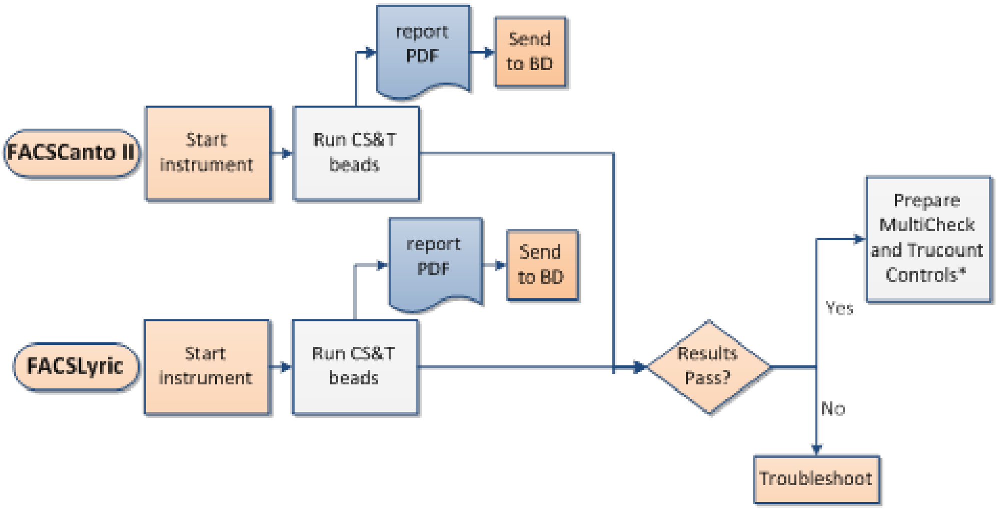

Figure 2. Process and Trucount Control Preparation and Acquisition

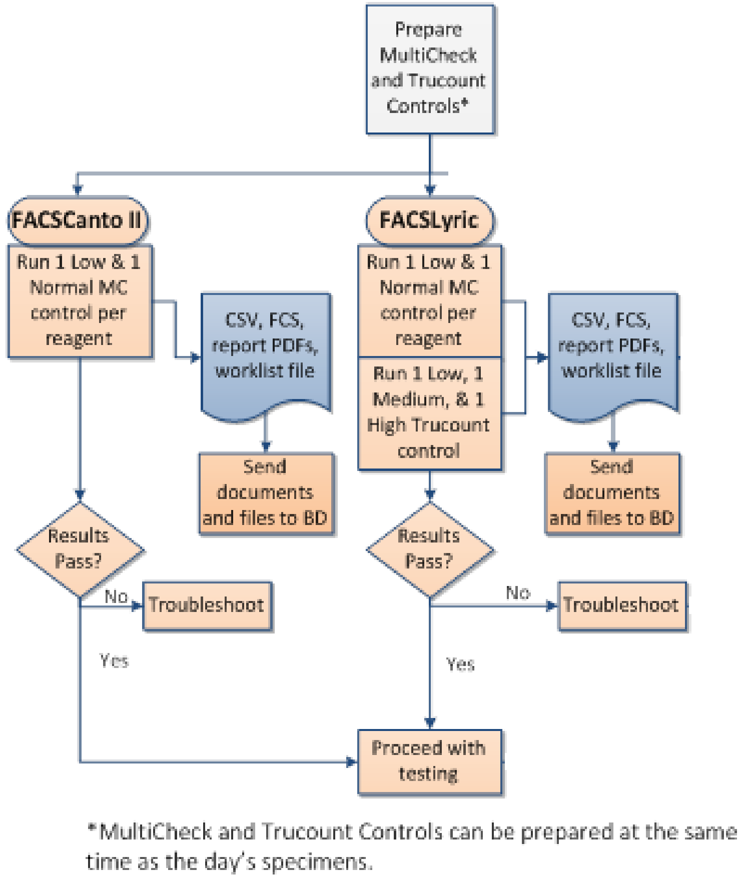

6.4 Inter-Laboratory Reproducibility: Sample Staining and Acquisition

Inter-laboratory reproducibility will be performed twice per day for a minimum of five non-consecutive days at three or more clinical study sites. Each day will include two runs of three replicates from a *specific lot* of ILR specimens. All clinical study sites will use the same lot of testing material. At least five non-consecutive days of evaluable data is required. Sites may need to capture data on additional days in order to meet this minimum. The steps for Inter-Laboratory Reproducibility testing are depicted in Figure 3.

Figure 3. Inter-Laboratory Reproducibility Testing

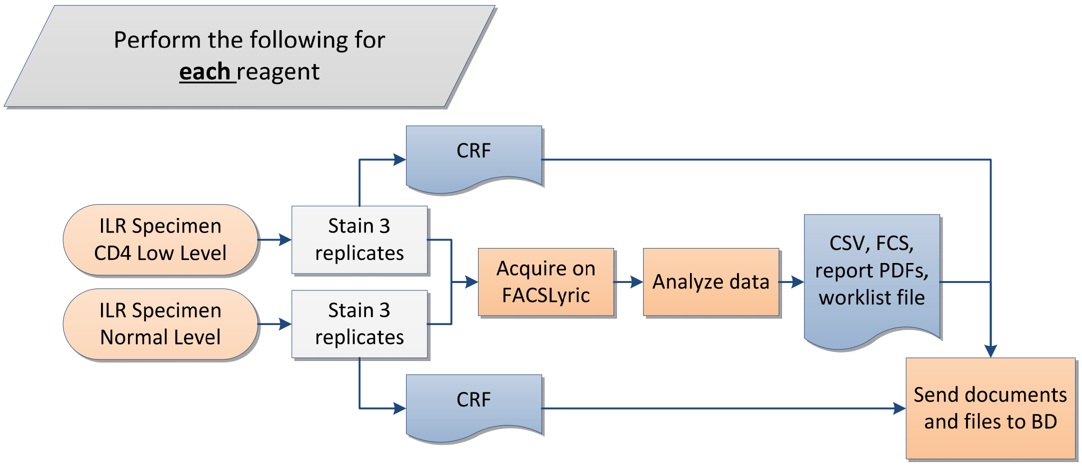

During each of two runs of testing per day:

- Stain 3 replicates of each ILR specimen (Normal and CD4 Low levels) with the assay reagents for the Sub-Study being tested (BD Multitest 6-color TBNK and BD Multitest IMK kit). Label tubes accordingly. Complete CRFs.
- Acquire stained controls on the IUO BD FACSLyric after setup and process controls, and within the age of stain indicated for the assay.

6.5 Sample Staining and Acquisition

Initially, an enrolled specimen should have enough volume to be used for the study. As CD4 bins are filled for each Sub-Study, sponsor will inform site that testing is no longer necessary for specific assay or Sub-Study. The steps for Method Comparison testing are depicted in Figure 4.

Figure 4. Method Comparison Testing

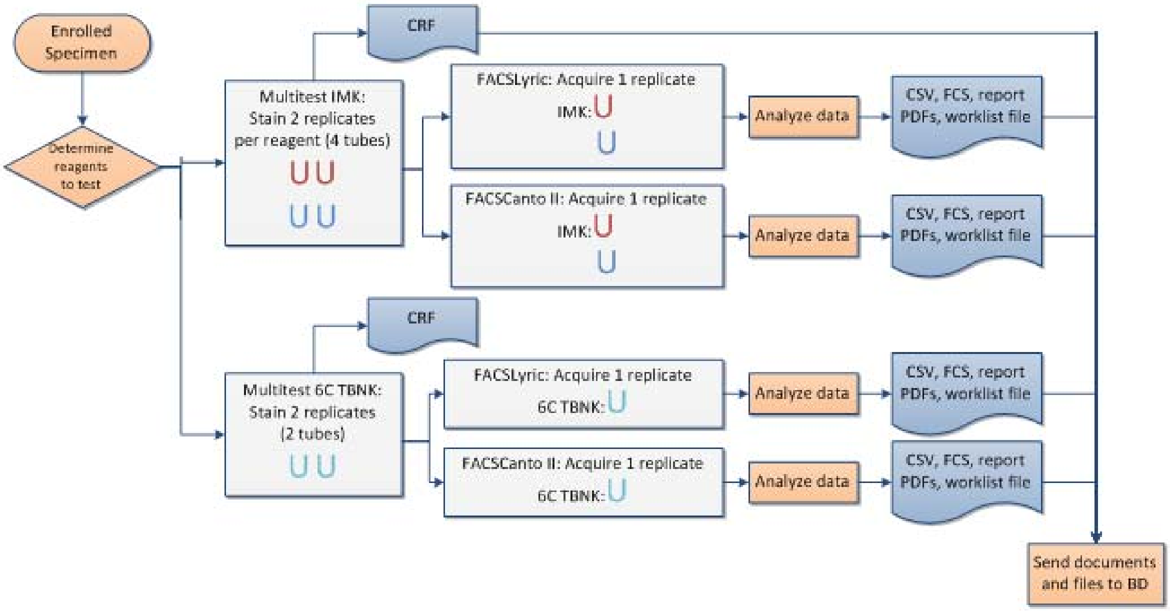

Specimen should be stained with Multitest IMK kit and Multitest 6-color TBNK reagents.

- For each enrolled specimen, stain the specimen in singlicate for each instrument and complete CRFs:
  - Stain two tubes with BD Multitest 6-color TBNK reagent using Trucount Tubes. (Total of 2 tubes.)
  - Stain two tubes with each reagent in the BD Multitest IMK kit using Trucount Tubes. (Total of 4 tubes.)
  - All tubes should be stained within 2h of each other.
- Acquire the data from one replicate on the IUO BD FACSLyric and acquire the other replicate on the FACSCanto II. Data from all replicates should be collected within 5h of each other.

If there is an issue with the data acquisition of a tube, rerun the tube. If there is still an issue, stain a new tube and acquire data from it. Appropriate CRF(s) must be completed.

6.6 Data Review and Analysis

- After data acquisition, all data from the investigational and predicate instruments should be reviewed and gates adjusted, as necessary. Store electronically or print all reports. Export CSV files.
- Review documentation and prepare data packet, to include electronic instrument data files, PDFs, completed CRFs, etc.

- Store data on provided electronic devices and in data binders.
- For submission of data to BD, print outs and CRFs will be scanned into electronic files. Provide data package to BD using the BD format transfer protocol (FTP) site or, if required, by mail.

6.7 Required Training and Proficiency Testing

All study staff participating in the study will be trained in the study design and study procedures. Training will be documented. Each operator must also pass proficiency evaluation. Proficiency testing will involve performing the study workflow, including instrument startup, processing and running of controls and specimens, completing all appropriate paperwork, and submission of data. The data will be compared to expected criteria, and paperwork (CRFs, etc.) will be evaluated, for determination of the operator passing. Instructions for proficiency testing will be provided prior to study execution.

6.8 Customer Ease of Use/Usability

The IUO BD FACSLyric System will be evaluated after the operator(s) at the site(s) have completed Proficiency Training and before the start of enrollment. The operators will complete and return the Eases of Use survey to BD.

- Ease of use survey will consist of questions addressing workflow and functionality of the IUO BD FACSLyric System. Each question will have 6 possible answers with a numeric value assigned for analysis. Example of the answers as the following:
  - Extremely easy/satisfied, numeric value = 5
  - Somewhat easy/satisfied, numeric value = 4
  - Neutral, numeric value = 3
  - Slightly difficult/dissatisfied, numeric value = 2
  - Very difficult/dissatisfied, numeric value = 1
  - Not applicable (NA), numeric value = 0

6.9 Additional Site Responsibilities

6.9.1 IUO BD FACSLyric Monthly Maintenance

The site will be responsible for normal monthly maintenance of the IUO BD FACSLyric flow cytometer. On a monthly basis, the "monthly clean procedure" needs to be performed. Instructions are in the Instructions for Use. Documentation of cleaning needs to be sent to BD.

Also, BD FC beads need to be run every month to set compensation controls in order for study data to be evaluable. Documentation of running the BD FC beads needs to be sent to BD.

6.9.2 Predicate Instrument Maintenance

Instruments should be cleaned on a monthly basis or as indicated in site protocols.

6.9.3 Communication with BD

As necessary, the site will be required to respond to DCFs and other study related questions in a timely fashion. Delay in responding could result in data being considered non-evaluable.

7.0 INTERRUPTION OR DISCONTINUATION OF PARTICIPATION/TESTING

7.1 Discontinuation of Specimen Testing

Post-enrollment, instances may occur that will require the discontinuation of the specimen testing such as:

- If further visual inspection of an enrolled specimen reveals clotting and/or hemolysis that was not initially apparent, this specimen would be unsuitable for study testing or, if results have been generated, these results would not be included in analysis.
- If an enrolled specimen (of sufficient quantity) is broken/spilled, this "quantity not sufficient (QNS)" specimen would require discontinuation of study testing.
- If an enrolled specimen (with adequate time post-draw to enroll for study testing) cannot be stained within the allotted time due to unanticipated testing difficulties, this specimen would require discontinuation of study testing. The decision to discontinue testing of a specimen can be made for each Sub-Study.

7.1.1 Replacement of Discontinued Subjects/Specimens

Discontinued samples will be replaced by additional enrolled specimens.

7.1.2 Retention of Data from Discontinued Subjects/Specimens

No data will be collected from subjects/specimens after the point of discontinuation except as needed to follow ongoing adverse events. All study data collected from the subject up to the point of discontinuation will be recorded on the Case Report Form, entered into the study database, and included in subsequent analyses, as appropriate.

8.0 RISK / BENEFIT ASSESSMENT

8.1 Potential Risks

Sites will use only remnant specimens for the execution of this protocol. Therefore there are no risks to the patient/subject. In addition, the results produced for this study:

- Will not be used for diagnosis or treatment of the patient/subject;
- Will include parallel testing by an approved and established method.

To reduce the likelihood of any risks to site staff participating in this investigation, all processing, and testing of potentially infectious Specimens must always be performed according to Standard Precautions, CDC Guidelines, Standard Guidelines, and the participating sites' standard operating procedures and policies.

8.2 Potential Benefits

There are no direct benefits to the subject for participation in this study.

9.0 ASSESSMENT OF SAFETY AND ADVERSE EVENTS

This study is being conducted with remnant specimens and does not include any human subjects. Refer to Study Incidents (section 10.0) for procedures in the event of injury to study site personnel.

10.0 INCIDENTS

A Clinical Study Incident is defined as any problem or issue involving the investigational product(s), reference methods, associated procedures or equipment, or represents a product-related injury (or potential for injury) to study subjects or personnel as a result of execution of this protocol. Clinical Study Incidents may adversely (or potentially adversely) affect human safety, the integrity of the evaluation data, or the operation of devices or systems, and warrant prompt attention.

Examples of Clinical Study Incidents that are not Adverse Events might be **mislabeling or adulteration of the investigational device, equipment or device malfunctions, errors in the device instructions, damage to devices caused by shipping or handling or improper storage, or injury to study personnel due to execution of the protocol**. If appropriate, an Incident may also be documented and reported as a protocol deviation.

Study-specific procedures for reporting Incidents, as well as adverse events and protocol deviations, will be provided to the study site prior to study execution. The Monitor should be contacted immediately when site becomes aware of or suspects any defective or malfunctioning product. This includes:

- Products that are involved in Study Incidents,
- Products that are found to be expired, damaged or defective,
- Products that are possibly the cause of an adverse effect, regardless of whether the product was believed to be damaged, defective or malfunctioning.

Such products (whether investigational or marketed) should be segregated and returned with appropriate documentation to the BD address below, unless instructed otherwise by BD. The Study Monitor should be contacted with any questions regarding return of study products. BD will supply mailing kits specifically intended for product contaminated with potentially bio-hazardous material.

11.0 RETURN OR DESTRUCTION OF STUDY PRODUCT

All disposable, used products not failed, damaged or otherwise involved in an Incident or Adverse Event are to be discarded into appropriate waste containers at the investigational site.

Unless instructed otherwise by BD, the Investigator will return all remaining unused or unopened test, reference, and ancillary study products to BD. At the conclusion of the study, and as appropriate during the course of the study, any products, supplies or BD equipment that are required to be returned will be shipped to BD at the address below, unless instructed otherwise:

Study Manager
2350 Qume Drive
San Jose, CA 95131
(408) 954-2384

12.0 DATA COLLECTION AND MANAGEMENT

12.1 Source Documents

Source data includes all information in original records (and certified copies of original records) of clinical findings, observations, or other activities (in a clinical study) used for the reconstruction and evaluation of the study. Source data are contained in source documents (original records or certified copies) and are used to verify the authenticity of information recorded on the Case Report Form (CRF). Typical source

documents include the hospital chart, medical office file, laboratory report, clinician notes, patient record, recorded data from automated instruments or other documentation prepared and maintained by the investigator/staff or ancillary services which contains a record of all observations and other data pertinent to the investigation on a study subject.

The investigator is required to maintain original source documents at the site. Should an original source document (e.g., an instrument printout, direct entry CRF) need to be forwarded to BD for data entry, the site must retain a clearly designated certified copy. The Study Monitor will confirm that procedures for copy certification have been established at the site prior to transmittal of any original source documents.

12.2 Case Report Forms (CRF)

The case report forms (CRF) will be provided by the Sponsor. The term “CRF” as used in this protocol may refer to traditional paper CRFs, or electronic case report forms for electronic data capture (EDC), as determined by the Sponsor.

The Investigator may delegate CRF completion to study personnel. However, the Sponsor must be apprised in writing of the name of such persons and the scope of their authority. The Principal Investigator or designee is obligated to review each CRF page and sign or initial the indicated pages using ink or for EDC, an electronic signature.

All entries to a paper CRF should be made clearly in black or dark blue indelible ballpoint pen to ensure the legibility of self-copying or photocopied pages. Corrections are made by placing a single horizontal line through the incorrect entry, so that the original entry can still be seen, and placing the revised entry beside it. The revised entry must be initialed and dated by a member of the Investigator's research team authorized to make CRF entries. Correction fluid must not be used.

CRF entries will be compared to source documents by the study monitor or designated personnel. Unless specified otherwise, all information on the CRFs must be traceable to original source documents.

12.2.1 CRF as Source Document

Some study data will be collected directly on CRFs and there will be no matching source from which to verify the entry. As a result, these CRFs serve as original source documents.

12.2.2 CRF/Data Transmittal

CRF and other data should be transmitted to the sponsor within one week of the testing date. Instructions for CRF and Data Transmittal will be provided to the Investigator at Study Initiation. Specific procedures may be described in a study-specific Monitoring Plan.

12.3 Electronic Source Data (optional)

Electronic files generated from study instruments contain source data. The site must maintain electronic files of all these data on storage devices (CDs, USB drives, etc.) provided by the Sponsor.

Some electronic source data will need to be transmitted to the Sponsor within one week of the testing date. Instructions for Data Transmittal will be provided to the Investigator at Study Initiation

Specific procedures will be described in a study-specific Data Management Plan.

12.4 Data Management and Storage

Data Management will be performed by the Sponsor. Data from completed CRFs will be entered into a controlled database and the database verified for accuracy against the CRFs, when applicable. If electronic data capture is utilized, the electronic records entered at the site will be entered directly into the controlled

database. Data security is ensured through password protection, limited access, audit trails, and regular backups of the data. Upon completion of the study and verification of data, data will be screened for accuracy and completeness, after which the database will be locked from any additional changes. A copy of the locked database will be provided to the BD Corporate Statistics Department for statistical analysis.

13.0 STATISTICAL METHODS

13.1 Sample Size Determination

For Method Comparison study, each Sub-Study will have a minimum sample size of 240 evaluable specimens across all sites that provide valid results to determine the bias (expected difference) between the investigational system versus the predicate system.

Inter-Laboratory Reproducibility evaluation and analysis are based on the CLSI guideline EP05-A3[2]. In line with guidance from this document, evaluable data will be collected from 3 sites for a minimum of 5 non-consecutive days, 2 runs of 3 replicates on each day.

13.2 Data Evaluability

Data will be considered Evaluable if all the following is met:

- Adequate controls have been run and are acceptable.
- Data has been produced from testing of an evaluable specimen (has met inclusion and exclusion criteria).
- Specimen testing has occurred within the allotted time for the assay (Age of Stain).
- Results are within the reportable range of the assay.
- Data packets are complete and all forms are complete.
- Documentation is complete.

Discrepancies in source documents can result in the data being considered non-evaluable.

13.3 Statistical Methods

13.3.1 General Statistical Considerations

All statistical analyses will be performed using commercially available statistical software. Adequate source document verification and/or audit activities will be utilized to assure the validity of performance evaluation conclusions. Analysis shall be performed based on methods described in the Statistical Analysis Plan. All related and resulting reports, documents and data shall be produced and maintained in such a way as to ensure their control and the protection of subject privacy as far as is reasonably practicable. Data files and analytic reports will be archived according to requisite regulatory standards.

13.3.2 Method Comparison Statistical Methods

Evaluation of data will follow CLSI guideline EP09-A3[3]. Estimates from the Deming regression will be used to evaluate the equivalency between the investigational and predicate systems of the following parameters:

AbsCD3, AbsCD4, AbsCD8, AbsCD19, AbsCD16+CD56, %CD3, %CD4, %CD8, %CD19, %CD16+CD56.

(For Multitest IMK kit, AbsCD3 and %CD3 values from tubes for reagents A and B will be averaged.)

Additional analysis may be carried out for information only as depicted in the Statistical Plan.

Ease of Use data will be discrete and qualitative variables obtained from small number of individuals at the participating clinical sites in the study. Data will be summarize and presented in tables or graphs as part of Human Factors and Final Clinical Study Reports.

13.4 Demographics/Other descriptive information

This study will not generate demographics or other descriptive information.

13.5 Interim analysis

Interim analysis of the study data may be performed as needed.

14.0 QUALITY CONTROL AND ASSURANCE

14.1 Accountability of Study Products

Investigational study products will be only be released for use to Investigators who have obtained written IRB/EC approval (as required) for participation in this study, who have completed all required study documentation, and who have been qualified by the Sponsor. Investigators must maintain control over all study products, and ensure they are used in accordance with this protocol. Failure to do so may result in the Sponsor suspending or terminating the study at the Investigator's site.

The Investigator will ensure that study products are only dispensed to subjects (or used for specimens) properly enrolled in the study. The Investigator must maintain records of receipt, disposition, return and/or destruction of all study products. All investigational study products released to the site must be accounted for at the unit level prior to study close out, regardless of disposition. The Study Monitor will regularly review all records regarding study product accountability.

The Sponsor will maintain records that document the shipment, receipt, disposition, return and/or destruction of study products.

14.2 Monitoring

BD, the study sponsor, will designate trained and qualified personnel to monitor the progress of this clinical study in accordance with BD Monitoring SOPs and the study-specific Monitoring Plan. A pre-study site qualification visit will be conducted to assess the adequacy of the site facilities and staff with respect to study requirements.

Prior to study start, a study initiation visit will be conducted to provide training to site staff with regard to the protocol, the completion of study documentation and Case Report Forms (CRFs), the monitoring schedule, and all regulatory requirements. During the study, routine monitoring visits will be conducted to assure the site continues to adhere to the protocol, the investigator agreement, and regulations regarding conduct of clinical studies. Assessments will be made regarding the subjects' protection and safety, when relevant, as well as the quality, completeness, and integrity of the data. The Study Monitor will assist the investigative site with query resolution and will perform site close-out activities once all queries have been resolved.

Additional visits may be carried out depending upon site activity and performance. The Investigator must agree to the inspection of all study related records and give direct access to source documents for verification of data on CRFs.

The Investigator is responsible for ensuring that any site-owned equipment required for use in the study is properly installed and maintained (e.g., inspected, calibrated, alarmed). Documentation of equipment maintenance procedures must be available for review by the Monitor.

14.3 Audits and Inspections

If the study is selected for audit by the Sponsor or if there is an inspection by the appropriate Health Authorities, then the Investigator and his team will make themselves available during the visit. The Investigator must agree to the inspection of all study related records and give the auditor/inspector direct access to source documents for verification of data on CRFs. The subject's anonymity must be safeguarded and data checked during the audit remain confidential.

As soon as the Investigator is aware of an upcoming inspection/audit by the Health Authorities, he/she will promptly inform BD. As agreed with the Investigator, BD personnel may be present at the site during the inspection.

14.4 Protocol Deviations

Protocol deviations are not permitted and should be implemented prospectively as a protocol amendment whenever practical or appropriate, unless required to protect the safety and well-being of the subject. The Investigator must notify the Sponsor immediately of any such deviation resulting from the need to protect a subject.

Protocol deviations (other than those required to protect the safety and well-being of a subject) may impact the evaluability of study data, and may place subjects at risk. If the Investigator or their staff inadvertently deviates from the study plan, the Investigator should implement appropriate corrective and preventive procedures, and should notify the Sponsor at their earliest convenience. Significant deviations may also need to be reported to the IRB/EC and local health authority.

The Study Monitor will evaluate records of study conduct at the site to identify any deviations, and will also report them to the Sponsor. Upon evaluation by the Sponsor, actions may be required to prevent additional deviations, such as retraining of the site, implementation of additional site procedures, and more frequent monitoring. If these steps fail, more serious measures, up to and including termination of the site and withdrawal of study product may be necessary.

15.0 ETHICAL AND REGULATORY STANDARDS

15.1 IRB/EC

An appropriate IRB/EC must review this protocol, the Informed Consent Form (if applicable), and any other supporting study documents which affect subject or study personnel safety, prior to study initiation at an investigational site. No investigational site may begin the study until the IRB/EC has given its written approval, signed by the IRB/EC chairperson or authorized personnel, and a copy of the approval letter and the approved Informed Consent Form (if applicable) has been provided to the Sponsor.

15.2 Informed Consent

For Primary Objective:

Only leftover de-identified/de-linked specimens from routine laboratory testing will be enrolled in the study and all subject's personal health information will be removed prior to enrollment. Therefore, Informed Consent may not be required by the IRB/EC.

For Secondary Objective:

Informed consent is not applicable for this study, as only commercial control materials are used for testing.

15.3 Confidentiality of Data

Subject confidentiality is strictly held in trust by the participating investigators, their staff, and BD and their agents. This confidentiality is extended to cover testing of biological samples in addition to the clinical information relating to participating subjects. Subject confidentiality and anonymity will be maintained at all times by removal of all identifiers from any data, clinical samples or documentation submitted for this study.

Any data collected meeting the definition of PHI will be collected and maintained using the designated authorizations and following all privacy procedures as specified in the applicable health authority regulations.

BD will maintain the security and confidentiality of all clinical study data sent to BD. BD clinical study databases will not be shared with any third party without the express written consent of the Principal Investigator and/or Site.

The Study Monitor or other authorized representatives of BD may inspect all documents and records required to be maintained by the Investigator. The Site will permit access to such records. BD and the Site may be required to provide regulatory agencies access to clinical study data and records, as well as source documents.

All other agreements as to confidentiality by BD, the Principal Investigator, and the Site may be found in the Confidential Disclosure Agreement and the Clinical Trial Agreement.

15.3.1 De-identification of Remnant Specimens

Personal information will be maintained confidential in accordance with the site's procedures and the Health Insurance Portability and Accountability Act (HIPAA) (if applicable). Donors/specimens will be assigned a coded identification number according to the site policy for delinked specimens. The coded list will remain with the clinical site or principal investigator or the site-specific policy will be followed.

For *in vitro* diagnostic device studies using leftover specimens, the FDA Guidance on Informed Consent for In Vitro Diagnostic Device Studies Using Leftover Human Specimens must be followed when Informed Consent is not obtained. The specimens must be provided to the investigator(s) without identifiers and the supplier of the specimens must have established policies and procedures to prevent the release of personal information.

15.4 Protocol Modifications

Amendments to the protocol will not be implemented without agreement from the Sponsor and prior submission to and written approval from the governing IRB/EC, except when necessary to eliminate an immediate hazard to the subject. Notice of an emergency modification shall be given to the Sponsor and the reviewing IRB/EC as soon as possible, but in no event later than 5 working days after the emergency occurred. Protocol amendments may affect Informed Consent Forms for current and future subjects.

Minor changes to the protocol, such as correction of typographical errors or changes in personnel names (other than the PI) or contact information will be processed as administrative changes. Administrative changes will be submitted to the governing IRB/EC but implementation of the administrative change may proceed without prior IRB/EC approval, unless so required by the IRB/EC or site SOPs.

15.5 Study Discontinuation

BD reserves the right to temporarily suspend or prematurely discontinue the study at a single site or at all sites at any time and for any reason. If such action is taken, BD will discuss the reasons with all Investigators (the Investigator). If the study is terminated or suspended due to safety reasons, the sponsor will inform the health authorities as required, and provide the reason(s) for the action. Investigator(s) must inform their IRB/EC promptly and provide the reason(s) for the suspension or termination.

15.6 Clinical Study Registration

In compliance with Title VIII of Public Law 110-85, known as FDA Amendments Act of 2007 (FDAAA), BD will register all applicable studies and disclose study results in a publicly accessible database, e.g. the ClinicalTrials.gov web site. Applicable studies will be registered no later than 21 days after commencing enrollment. Study results for applicable studies will be posted to the website within 12 months of the last subject visit for collection of primary outcome data, or after health authority approval for previously unapproved devices. BD has responsibility for determining whether this study qualifies as an “applicable” study under the law, and if so, will take responsibility for registration and disclosure as required by law.

15.7 Publication of Results

BD believes that results of applicable clinical studies of our products should be published in peer-reviewed literature in a timely, accurate, complete and balanced manner, regardless of study outcomes. BD is committed to making information public whenever it relates to the safety and efficacy of its marketed products.

Should this study be considered an “applicable study,” any formal presentation or publication of data collected from this study will be considered as a joint publication by the investigator(s) and the appropriate personnel of BD. Authorship will be based on generally accepted criteria of the ICMJE (International Committee of Medical Journal Editors) and determined by mutual agreement. For multi-center studies, it is mandatory that the first publication is based on data from all centers, analyzed as stipulated in the protocol by BD statisticians, and not based on data from single sites or a subset of sites. Investigators participating in multi-center studies agree not to present data gathered from one center or a small group of centers before the full, initial publication, unless formally agreed to by all other investigators and BD (the sole exception being an unanticipated adverse event that is product-related and which might have clinically significant safety implications for a marketed product or a class of products).

BD must receive copies of any intended communication in advance of publication as specified in the Clinical Study Agreement. In a timely manner, BD will review the communications for accuracy (thus avoiding potential discrepancies with submissions to health authorities), verify that confidential information is not being inadvertently divulged and to provide any relevant supplementary information to the investigators.

15.8 Record Retention

If the Principal Investigator or Clinical Center withdraws from the responsibility of keeping the study records, custody must be transferred to a person or entity who will accept the responsibility. BD must be notified in writing of the name and address of the new custodian.

Federal regulations require that a copy of all essential study documents (e.g., IRB/EC approvals, signed informed consent forms, source documents, CRF copies, safety reports, test article dispensing records, etc.), must be retained in the files of the responsible Investigator for a minimum of 2 years following notification

by BD that all investigations are completed, terminated, or discontinued, or that the FDA has approved the application (21 CFR 812.140).

16.0 BIBLIOGRAPHY/REFERENCES

1. E6, International Conference on Harmonization: Good Clinical Practice: Consolidated (Published in the Federal Register May 9, 1997).
2. CLSI EP05-A3: Evaluation of Precision of Quantitative Measurement Producres; Approved Guideline-Third Edition, October 2014.
3. CLSI EP09-A3: Measurement Producure Comparison and Bias Estimation Usng Patient Samples; Approved Guideline-Third Edition, August 2013.

17.0 PROTOCOL REVISION HISTORY

Version #	Rationale for Change	Section or Page affected	Description of change
1.0	New Protocol		
1.1	Administrative Change	*Throughout Document*	The inter-laboratory reproducibility testing was updated to allow CD Chex Plus controls or a custom lot of Multicheck controls to be the specimens used for testing.
2.0	Amendment	*Throughout Document*	Changed population to include specimens exhibiting leucopenia or leukocytosis in study, in addition to patients attending for HIV and immune reconstitution testing to and normal patients/donors. Rational: These assays are used to test immune status, not particular diagnoses.<br><br>Removed analysis of AveAbsCD3 of the reagents and requirement for stained samples to be run together. Rational: PI does not require to be run together<br><br>Administration changes/Correction of typos

18.0 APPENDICES

The following appendices are included:

- Study Design
- Instruments, controls, and reagents used, broken down by Sub-Study and step of study

18.1 Study Design

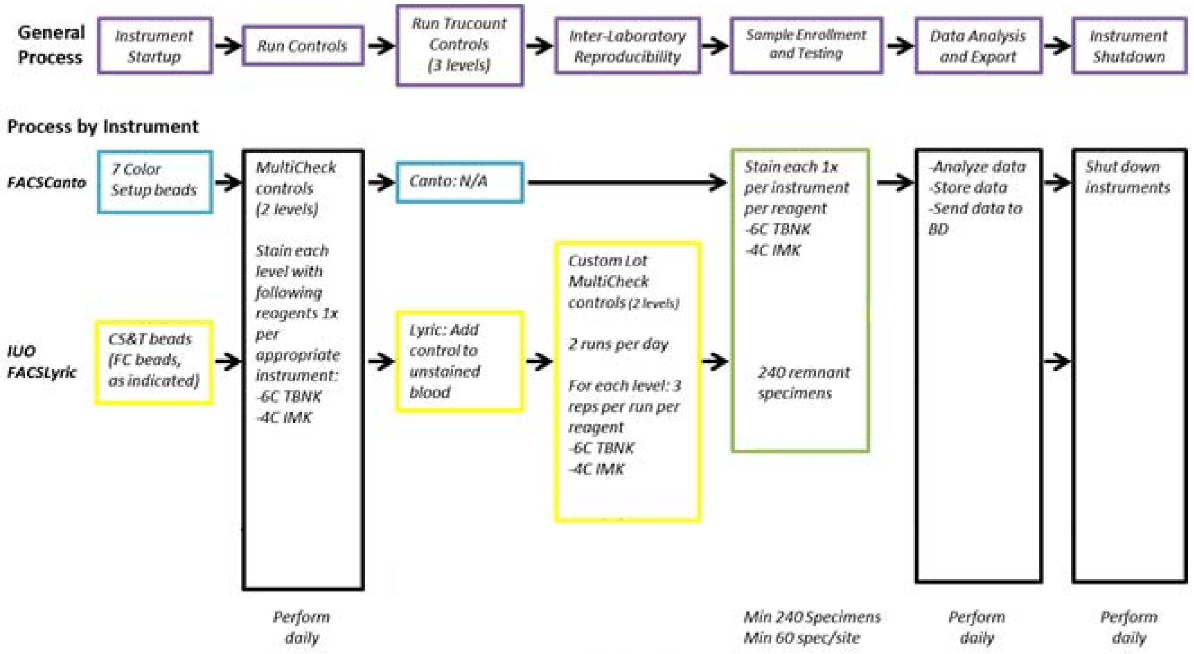

18.2 Instruments, controls, and reagents used, broken down by Sub-Study and step of study

**Investigational instrument, software, and setup reagents**
• IUO BD FACSLyric flow cytometer with IUO Universal Sample Loader<br>• Computer workstation with IUO FACSuite Clinical software<br>• IUO BD CS&T beads<br>• IUO BD FC beads (monthly)
**Predicate instrument, software, and setup reagents**
• IVD BD FACSCanto II with (optional) sample loader<br>• Computer workstation with FACSCanto Clinical software v2.4 or later<br>• IVD BD 7-Color Setup Beads
**Controls**
• BD Trucount Tubes<br>• BD MultiCheck (Normal and CD4 Low)
• BD Multitest 6-color TBNK<br>• BD Multitest IMK kit
**Inter-Laboratory Reproducibility (min 5 days, 2 runs of 3 reps per run, per day)**
• BD Multitest 6-color TBNK<br>• BD Multitest IMK kit
• ILR Specimens (Normal and CD4 Low levels)
**Testing Remnant Specimens**
• BD Multitest 6-color TBNK<br>• BD Multitest IMK kit
• BD Trucount Tubes
