# Supplementary material for: Accurate and reproducible enumeration of T-, B-, and NK lymphocytes using the BD FACSLyric 10-color system: A multisite clinical evaluation
Source: PLoS One. 2019 Jan 28;14(1):e0211207. doi: 10.1371/journal.pone.0211207 (PMC6349332; doi:10.1371/journal.pone.0211207)
Supplement: S2 File — (PDF) [file pone.0211207.s002.pdf]

Corporate Clinical Development
CCD-5115.T01 Protocol Template
Revision 04

BD Protocol #: **CAS-LIBHIVSTB**

Protocol Title: **Evaluation of the EDTA Anticoagulated Venous Blood and Specimen-Staining Stability Using the BD Multitest 4 and 6-Color Reagents on the Investigational BD FACSLyric 10-Color Clinical Flow Cytometer.**

**Current Version**	Version 2.0- 06/08/2016 (Rev 2.0)	
**Version History**	Version 1.0 -10/08/2015 (Original)<br>Version 1.1- 05/16/2016	
**Sponsor**	BD – BD Biosciences<br>2350 Qume Dr.<br>San Jose, CA 95131	
**Sponsor Risk Assessment**	☐ Significant Risk (SR)	☐ Non-significant Risk (NSR)<br>☒ Minimal Risk

The product information and data disclosed through this protocol are confidential and may not be disclosed without prior written consent of Becton, Dickinson and Company.

This study will be performed in accordance with all stipulations of the protocol and in compliance with all applicable BD Policies and Procedures. This study will be conducted in accordance with the ethical principles that originate from the Declaration of Helsinki and the Belmont Report. Study conduct will comply with US FDA Regulations, applicable state and local regulations, and the Good Clinical Practice guidelines set forth by the International Conference on Harmonization (ICH-E6).

The performance characteristics of this product have not been established. Results must not be used as a diagnostic procedure without confirmation of the diagnosis by another, medically established diagnostic product or procedure.

All Study Product(s) used in this study should be considered to be for investigational use only.

SPONSOR PROTOCOL APPROVAL

Signature below indicates approval of the protocol as written.			
**Individual or function**	**Name**	**Signature**	**Date**
Medical Affairs Team Representative		*This document is signed electronically in the eTMF system*	
Study Statistician		*This document is signed electronically in the eTMF system*	
Study Manager		*This document is signed electronically in the eTMF system*	
Regulatory		*This document is signed electronically in the eTMF system*	
Business Unit Medical Director		*This document is signed electronically in the eTMF system*	

INVESTIGATOR SIGNATURE PAGE

**Principal Investigator**	{Name}<br>{Address} (if different from site)
**Investigational Site**	{Site Name}<br><br>{Address}

I have read this protocol and agree to conduct this study in accordance with all stipulations of the protocol and in compliance with all applicable Good Clinical Practices and regulations.

____ ____________

Signature of Principal Investigator Date

Table of Contents

LIST OF ABBREVIATIONS AND DEFINITION OF TERMS 6

1.0 INTRODUCTION 7

2.0 OBJECTIVES 7
- 2.1 Primary Objectives 7
- 2.2 Secondary Objectives 8
- 2.3 Exploratory Objectives (if applicable) 8

3.0 STUDY DESIGN 8
- 3.1 Overall Study Design 8
- 3.2 Specification of Study Endpoints 8
- 3.3 Acceptance Criteria 9
- 3.4 Treatment Allocation and Methods to Reduce Bias 9
- 3.5 Stopping Rules 9

4.0 STUDY POPULATION 9
- 4.1 Inclusion Criteria 9
- 4.2 Exclusion Criteria 10

5.0 DESCRIPTION OF STUDY PRODUCTS 10
- 5.1 Test Product(s) 10
- 5.2 Reference Products (or Methods) 11
- 5.3 IVD Reagents 11
- 5.4 Ancillary Products (if applicable) 11
- 5.5 Product Labeling 11
- 5.6 Maintenance and Storage of Study Products 12

6.0 STUDY METHODS 12
- 6.1 Summary of Daily Study Activities 12
- 6.2 Specimen Enrollment 12
- 6.3 Instrument Setup and Process Control 12
- 6.4 Sample Staining and Acquisition 13
- 6.5 Data Review and Analysis 16
- 6.6 Required Training and Proficiency Testing 17
- 6.7 Customer Ease of Use/Usability 17
- 6.8 Additional Site Responsibilities 17
- 6.9 Communication with BD 17

7.0 DISCONTINUATION OF TESTING 18
- 7.1 Discontinuation of study specimens 18
- 7.2 Replacement of Discontinued Specimens 18
- 7.3 Retention of Data from Discontinued Specimens 18

8.0 RISK / BENEFIT ASSESSMENT 18
- 8.1 Potential Risks 18
- 8.2 Potential Benefits 18

9.0 ASSESSMENT OF SAFETY AND ADVERSE EVENTS 18
9.1 Adverse Event Definitions 18
9.2 Adverse Event (AE) Management 19
9.3 Assessment of Adverse Events (AEs) 20
9.4 Additional procedures for Assessing & Reporting Serious Adverse Events (SAE) 20
10.0 INCIDENTS 21
11.0 RETURN OR DESTRUCTION OF STUDY PRODUCT 21
11.1 Source Documents 22
11.2 Case Report Forms (CRF) 22
11.3 Electronic Source Data (optional) 23
11.4 Data Management and Storage 23
12.0 STATISTICAL METHODS 23
12.1 Sample Size Determination 23
12.2 Data Evaluability 23
12.3 Statistical Methods 24
12.4 Demographics/Other descriptive information 25
12.5 Interim analysis 25
13.0 QUALITY CONTROL AND ASSURANCE 25
13.1 Accountability of Study Products 25
13.2 Monitoring 25
13.3 Audits and Inspections 26
13.4 Protocol Deviations 26
14.0 ETHICAL AND REGULATORY STANDARDS 26
14.1 IRB/EC 26
14.2 Informed Consent 26
14.3 Confidentiality of Data 26
14.4 Protocol Modifications 27
14.5 Study Discontinuation 27
14.6 Clinical Study Registration 27
14.7 Publication of Results 28
14.8 Record Retention 28
15.0 BIBLIOGRAPHY/REFERENCES 28
16.0 PROTOCOL REVISION HISTORY 28

LIST OF ABBREVIATIONS AND DEFINITION OF TERMS

AE	Adverse event
AOB	Age of blood
AOS	Age of stain
BD	Becton Dickinson and Company
CFR	Code of Federal Regulations
CI	Confidence Interval
CRF	Case Report/Record Form
DCF	Data Clarification Form
EDC	Electronic Data Capture
EDTA	Ethylene diamine tetra acetic acid
FDA	Food and Drug Administration
FDAAA	FDA Amendments Act of 2007
GCP	Good Clinical Practice
HIPAA	Health Insurance Portability and Accountability Act
ICH	International Conference on Harmonization
IFU	Instructions for Use
IRB/EC	Institutional or Independent Review Board/Ethics Committee
IUO	Investigational Use Only
IVD	In Vitro Diagnostics
LIS	Laboratory Information System
µL	microliter
mL	Milliliter
mm	Millimeter
PEO	Performance Evaluation Only
PI	Package Insert
QNS	Quantity Not Sufficient
SAE	Serious Adverse Event
SD	Standard deviation
SOP	Standard Operating Procedure
WFN	Workflow Notes
WHO	World Health Organization

1.0 INTRODUCTION

The IUO BD FACSLyric system is a clinical analyzer. At launch, it is intended to meet the needs of the routine clinical flow cytometry markets performing lymphocyte subset identification and enumeration of human cells in suspension. The applications will later be expanded for a broader market segment including blood bank screening, Stem Cell enumeration, and Activation Marker.

The IUO BD FACSLyric system will serve as a replacement for the instrument/software systems of BD FACSCalibur/FACSComp and Multiset software and BD FACSCanto II/Canto Clinical software as the "workhorse" system.

The IUO BD FACSLyric system will be cleared as an IVD system (cytometer/reagents/software/options) with BD Bioscience's currently marketed IVD assays and process controls. This clinical stability study supports US 510(k) submission and CE-IVD application.

The IUO system consists of the IUO BD FACSLyric 10 color flow cytometer with Universal Loader, a computer workstation with the IUO FACSuite Clinical software, the use of the IUO BD CS&T Beads/IUO BD FC Beads and the FACSLink system.

For this study, four reagents/kits will be used for testing: BD Multitest 6-color TBNK, and the BD Multitest IMK kit. The BD Multitest IMK kit includes two reagents: BD Multitest CD3/CD8/CD45/CD4 reagent and BD Multitest CD3/CD16+56/CD45/CD19 reagent.

2.0 OBJECTIVES

2.1 Primary Objectives

The primary objective is to generate product claim data for stability of venous whole blood with EDTA anticoagulant using the investigational-use-only (IUO) labeled system (IUO BD FACSLyric/IUO FACSuite Clinical software/IUO BD FC beads/IUO CS&T beads) using the following previously cleared IVD reagents:

- Multitest 6-color (6C) TBNK reagent with Trucount tubes for determination of lymphocyte sub-populations [absolute CD3+ (**AbsCD3**), CD3+CD4+ (**AbsCD4**), CD3+CD8+ (**AbsCD8**), CD16+CD56+ **(AbsCD16+56)**, and CD19+ **(AbsCD19)** cell counts, and percentage of CD3+ (**%CD3**), CD3+CD4+ (**%CD4**), CD3+CD8+ (**%CD8**), CD16+CD56+ **(%CD16+56)**, and CD19+ **(%CD19)**] using prospectively procured and de-linked patient specimens from at least two study sites.
- Multitest IMK kit [4-color (4C)] with Trucount tubes for determination of lymphocyte sub-populations [absolute CD3+ (**AbsCD3**), CD3+CD4+ (**AbsCD4**), CD3+CD8+ (**AbsCD8**) CD16+CD56+ **(AbsCD16+56)**, and CD19+ **(AbsCD19)** cell counts, and percentage of CD3+ (**%CD3**), CD3+CD4+ (**%CD4**), CD3+CD8+ (**%CD8**), CD16+CD56+ **(%CD16+56)**, and CD19+ **(%CD19)**] using prospectively procured and de-linked patient specimens from at least two study sites.
- \

2.2 Secondary Objectives

Evaluate the customer's ease of use of the IUO BD FACSLyric/ FACSuite software/BD FC and CS&T beads.

2.3 Exploratory Objectives (if applicable)

NA

3.0 STUDY DESIGN

3.1 Overall Study Design

At two or more sites, prospective evaluation of the investigational BD FACSLyric flow cytometer uses freshly procured venous whole blood specimens tested at the reagent specific acquisition time points. The prospectively enrolled venous whole blood specimens will be obtained from patients attending clinics for routine HIV or immune reconstitution testing or from healthy donors. The specimens enrolled must allow sufficient time to stain the specimen within 6 h of blood draw and sufficient volume for testing. Based on the FDA feedback from June 15, 2015, the study design has incorporated the suggested subject enrollment to support stability testing for age of blood and age of stain relative to the cleared product claims specific for each reagent. It is expected that up to 500 specimens will be enrolled to satisfy the time point bins and CD4 bins, specifically for the CD4+ cells in the lower range of <200 cells/µL for all reagents, as described in section 12.3. Detailed explanation of the reagent time points can be found in section 6.4.

- **Time Points** refers to the reagent specific staining and testing times:
- **Age of blood** (AOB) refers to the time between the blood draw and the time of mixing the blood with the reagent(s).
- **Age of stain** (AOS) refers to the time between the end of the lysing step and the start of specimen acquisition.
- **Time Zero** or reference result refers to the first time point collected for a specimen, which must be stained within 6 hours of the draw, and must be acquired within 6 hours of stain (T0/T0). This time point is required for the specimen to be evaluable. This time point is referred to as the Reference T0/T0.

Enrolled specimens may be tested by one or more reagents depending on the venous whole blood volume and the amount of time available to the operator to complete the testing. Workflow and time points will be discussed in further detail in Section 6: Study Methods.

3.1.1 Study Duration

The expected duration from start to the completion of the stability evaluation is anticipated to be 1-4 months, including site training and proficiency testing.

3.2 Specification of Study Endpoints

3.2.1 Primary Endpoints

The primary endpoint of the study is to generate the data supporting the product claims for stability of the EDTA venous blood and the specimen staining during storage for the Multitest 6 color TBNK

reagent, and Multitest IMK Kit, reagents on the IUO BD FACSLyric System from a minimum of 90 prospectively procured specimens obtained.

3.2.2 Secondary Endpoints

The customer's response on the "ease of use" survey is a qualitative assessment of how easily and safely an instrument operator can use the IUO BD FACSLyric System.

3.2.3 Safety Endpoints

During study testing, occurrence and severity of any adverse events will be evaluated, recorded, and followed up as required.

3.3 Treatment Allocation and Methods to Reduce Bias

The venous whole blood specimens will be enrolled in this study anonymously in a random order and tested for each of the reagents as allowed by the available volume of the blood specimen and operator time.

3.4.1 Skill and Behavior of Persons Interacting with the Device (if applicable)

The operators performing this assay are skilled laboratorians either in the area of flow cytometry or in instrumented assay expertise. They will be required to verify their skill set by passing proficiency prior to execution of the study requirements.

3.4 Stopping Rules

When using specimens, no stopping rules for the study have been developed by the Sponsor. The Principal Investigator is responsible for suspending study enrollment for reasons of subject/clinician safety and well-being.

Specimens will be prospectively collected for this study. If the phlebotomy procedure were determined to pose an issue that could potentially affect the health of the subjects, then the Principal Investigator is responsible for stopping the blood collection.

4.0 STUDY POPULATION

Unless specified otherwise, these criteria apply at specimen screening and throughout the study. This study will be conducted at a qualified clinical laboratory. Study site(s) will enroll venous whole blood specimens that are post routine testing in the lab or that are prospectively enrolled for the purpose of this study. These specimens will be de-linked and enrolled into the study for stability testing.

4.1 Inclusion Criteria

Specimens that satisfy all of the following conditions will be considered for participation:

a. Specimens are collected from subjects willing to participate and provide written informed consent

b. Whole blood is used as the specimen and is collected in accordance with site's policies and procedures.

c. The whole blood is drawn into EDTA anti-coagulant.

d. The specimen draw date and time is provided.

e. The specimen has sufficient volume to perform the staining and acquisition as required for the stability time points. (approximately 1.5 mL)

f. The specimen is available with time for enrollment and sample processing within 6 hours of collection.

g. The specimen is stored at 20 ºC to 24 ºC from the time of collection through enrollment and until data from all time points for the specimen have been acquired and analyzed.

4.2 Exclusion Criteria

Specimens with any one of the following characteristics will be excluded from participation in this study:

a. Clotted or hemolyzed specimens
b. Frozen or refrigerated specimens or samples
c. Fixed samples

Enrolled specimens may be subsequently excluded from study testing if unsuitable for testing, for example, visual inspection prior to sample acquisition shows clotting or hemolysis. See Section 7.0: Discontinuation of Testing for further information.

5.0 DESCRIPTION OF STUDY PRODUCTS

The BD FACSLyric flow cytometer and FACSuite Clinical software with IVD assays, including optional Universal Loader and LIS options (FACSLink), will be a replacement instrument/software system for the current BD FACSCalibur/ FACStation software system as well as the BD FACSCanto II/ BD Canto Clinical software.

5.1 Test Product(s)

- IUO labeled BD FACSLyric 10 color flow cytometer with 4-3-3 configuration (BD Catalogue # 659180)
- FACSLyric Universal Loader (BD Catalogue # 651166)
- IUO BD FACSuite workstation
- IUO FACSuite Clinical software and dongle
- IUO Multitest assay module
- IUO 6C TBNK assay module
- IUO BD CS&T beads (BD Catalogue # 656504/656505)
- IUO BD FC beads (7-Color Kit for BD FACSuite)
- IUO FACSLyric Instructions for Use
- IUO FACLyric Safety and Limitations Guide
- IUO Assay Setup Report
- IUO QC Report
- IUO Lab Report
- IUO Physician Report

- IUO Supplemental Report

5.2 Reference Products (or Methods)

There are no reference products for this study.

5.3 IVD Reagents

- BD Multicheck Normal process controls (BD Catalogue # 340911, 340912, 340913)
- BD Multicheck CD4 Low process control (BD Catalogue # 340914, 340915, 340916)
- BD Multitest 6-color TBNK reagent (BD Catalogue # 644611)
- BD Multitest IMK Kit (BD Catalogue # 340503)
- BD Trucount Tubes (BD Catalogue # 340334)
- BD FACS Lysing Solution (BD Catalogue # 349202)

5.4 Ancillary Products (if applicable)

BD will provide the following products as needed.

- Electronic pipette(s) (BD Catalogue # 646539 or similar)
- External Hard Drives or USB memory sticks for archival of the instrument electronic files.
- BD FACSFlow sheath fluid (BD Catalogue # 342003)

Site should have access to the following:

- Calibrated pipettes and pipette tips (to dispense 20-450 µL)
- Vortex mixer
- Lab timer
- Clock
- Scanner
- Bleach
- Printer and related supplies
- Refrigerator with controlled temperature range between 2-8°C
- Disposable 12X75mm BD Falcon Capped polystyrene 5 mL tubes

5.5 Product Labeling

Investigational products (including marketed products used off-label, if applicable) and units must be labeled as “For Investigational Use Only. The performance characteristics of this product have not been established."

The investigational products for this study are the IUO labeled FACSLyric flow cytometer with universal loader, IUO FACSuite Clinical software with associated reports, IUO CS&T beads, and IUO FC beads.

For investigational products, labeling will include the following at minimum, as applicable:

- Study Number
- Product Identification
- Manufacturer name and location
- Batch #/Lot #

- Use by/expiration date

Commercially available products used as intended will be supplied as labeled by the manufacturer.

5.6 Maintenance and Storage of Study Products

The BD FACSLyric instrument system and IUO FACSuite Clinical software shall be maintained at 16 - 29 ^{0}C. The IUO CS&T beads and IUO FC beads shall be stored at 2-8 ^{0}C. The BD Multitest IMK kit and BD Multitest 6-color TBNK reagent, and BD Multicheck process controls shall be stored at 2-8 ^{0}C. The BD Trucount tubes shall be stored at 2-25 ^{0}C. BD FACSLysing Solution and BD FACSFlow sheath fluid should be stored at 16-29˚C.

Additional instructions regarding product disposition during and upon study completion (e.g., disposal, return or destruction, defective products) will be discussed at training.

6.0 STUDY METHODS

6.1 Summary of Daily Study Activities

Each day's work will include completing relevant CRFs during the process.

- Enroll specimens that meet criteria (volume, time, other inclusion/exclusion criteria, informed consent), and re-label with Study ID number.
- Start-up instrument and perform instrument setup using appropriate beads. Confirm that instrument passes.
- Stain MultiCheck control and acquire on instrument in accordance with the manufacturer's instructions. Analyze data to determine if they are within manufacture's ranges.
- Stain samples as needed for the time point(s) to be tested. Acquire samples at appropriate time(s).
- Review and analyze study specimen data. Re-analyze, re-run, or re-stain as necessary.
- Store data on site and provide to BD.

6.2 Specimen Enrollment

BD Multitest IMK, and BD Multitest 6 color TBNKtesting requires prospectively procured venous whole blood EDTA samples of sufficient quantity from patients attending for routine HIV or immune reconstitution testing, or from healthy donors giving informed consent.

For all specimens,

- Confirm that specimen satisfies inclusion/exclusion criteria.
- De-identify specimen and assign a Study ID number. The site will maintain a coded list that associates the specimen identity with the Study ID number. This list will not be transferred to the sponsor and the operator running the test will not be the person who performs this de-identification.

6.3 Instrument Setup and Process Control

IUO BD FACSLyric:

- Start the instrument and workstation. Allow the instrument to warm up for 20 minutes prior to running samples or controls.
- In the IUO FACSuite software, run Performance QC with CS&T beads. Confirm that the instrument passed. Save report.
- Stain MultiCheck process controls (2 levels) with each of the reagents. Complete the CRFs. Controls may be stained at the same time as the test specimens.
- Acquire data on the controls. Review and analyze data to confirm measured values are within manufacturer's ranges prior to acquiring patient samples.

6.4 Sample Staining and Acquisition

An enrolled specimen should have enough volume to be used for testing by at least one of the three reagent sets. One specimen can be stained with more than one reagent if the specimen volume and technician time permits. The sample staining time points per reagent are discussed below for testing Day 1 through Day 4, as applicable. Please see Tables 1 through 4 for the specific details.

Sample preparation includes the following:

- For each time point for each reagent, mix the specimens well by gently inverting the EDTA blood tube a minimum of 10 times prior to dispensing a sample in duplicate Trucount tubes for each reagent to be tested.
- Add the reagent(s), then vortex and incubate the tubes in the dark for 15 minutes at room temperature. Follow this with the addition of the lysing solution, mixing of the samples, and a second 15 minute room temperature incubation in the dark. An enrolled study specimen will be acquired at all the required time points for the appropriate reagent following the instructions provided in the workflow notes.

Samples will be acquired according to the appropriate reagent time point matrix. The time point testing matrix per reagent includes the following assumptions:

- All reagents must collect time zero data.
- Time point(s) claimed in reagent package insert are marked as red X.
- Time point beyond claim in the reagent package insert is in the orange cell. The gray cells are not applicable for the reagent being tested.

6.4.1 Multitest IMK kit workflow

The BD Multitest IMK kit includes 2 reagents: BD Multitest CD3/CD8/CD45/CD4 and BD Multitest CD3/CD16+56/CD45/CD19. Testing for all time points will require 3 days, with two time points tested the last day (See Tables 1 and 4 below).

<u>Day 1:</u>

- Stain the specimen within ≤6h of blood draw in duplicate for each reagent being tested using 2 Trucount tubes for each reagent (total of 4 tubes). Complete the CRFs.
  - Acquire the data from one replicate on the IUO BD FACSLyric within ≤6h of staining. The appropriate CRF must be completed.

- o Review and approve results; if there is an issue with the data acquisition of a tube, rerun the tube. If there is still an issue, acquire the second tube ≤6h of staining. The appropriate CRF must be completed.
- o If there is still an issue with the data acquisition the specimen may be stained and acquired again, providing the AOB is ≤6h old when the staining occurs. Acquire the second set of specimens as needed. The appropriate CRF must be completed.

Day 2:

- Stain the specimen which is now ≥23/≤25h of draw using 2 Trucount tubes for each reagent (total of 4 tubes). Hold the stained tubes in the dark at room temperature for 23-25h.
  - o Acquire the data from one replicate for each reagent on the IUO BD FACSLyric within 23-25h of staining.
  - o Review and approve results; if there is an issue with the data acquisition, rerun the tube. If there is still an issue, acquire the second tube within 23-25h. The appropriate CRF must be completed.

Day 3 (1st stain):

- Stain the specimen which is now ≥47/≤49h of draw using 2 Trucount tubes for each reagent (total of 4 tubes). Hold the stained tubes in the dark at room temperature for 23-25h.
  - o Acquire the data from one replicate for each reagent on the IUO BD FACSLyric within 23-25h of staining.
  - o Review and approve results; if there is an issue with the data acquisition of a tube, rerun the tube. If there is still an issue, run the other replicate. The appropriate CRF must be completed.

Day 3 (2nd stain):

- Stain the specimen which is now ≥50/≤52h of draw using 2 Trucount tubes for each reagent (total of 4 tubes). Hold the stained tubes in the dark at room temperature for 25-27h.
  - o Acquire the data from one replicate for each reagent on the IUO BD FACSLyric within 25-27h of staining.
  - o Review and approve results; if there is an issue with the data acquisition of a tube, rerun the tube. If there is still an issue, run the other replicate. The appropriate CRF must be completed.

Table 1. 4C-Multitest Testing Matrix

4C-MT		Claim 48 AOB /24 AOS			
		AOS (h)			
		≤6(0-6)	8(7-9)	24 (23-25)	26 (25-27)
AOB (h)	0-6	X	NA	NA	NA
	24 (23-25)	NA		X	

	48 (47-49)			X	
	51 (50-52)			NA	X

X	Time point
X	Claim
X	Time point after Claim

Table 2. Study Time Point for 4C-MT Reagents, Age of Blood and Age of Stain

No.	Time Point	Age of Blood (AOB)	Age of Stain (AOS)
1	T0 /T0	Reference	
		≤6h	0-6h
2	T24/T24	23-25h	23-25h
3	T48/T24	47-49h	23-25h
4	T51/T26	50-52h	25-27h

6.4.2 **Multitest 6-Color TBNK reagent workflow:**

Multitest 6-Color TBNK testing will be carried out over two days with two time points stained on the second day (See Tables 3 and 4 below).

Day 1:

- Stain the specimen in duplicate within ≤6h of draw using Trucount tubes and complete the CRFs.
    - Acquire the data from one replicate on the IUO BD FACSLyric within ≤6h of staining.
    - Review and approve results; if there is an issue with the data acquisition of a tube, rerun the tube. If there is still an issue, run the other replicate. The appropriate CRF must be completed.
- If there is still an issue with the data acquisition the specimen may be stained and acquired again, providing the AOB is ≤6h old when the staining occurs. Acquire the second set of specimens as needed. The appropriate CRF must be completed.

Day 2 (First Stain):

- Stain the specimen in duplicate within ≥23/≤25h of draw using Trucount tubes and complete the CRFs.
    - Acquire the data from one replicate on the IUO BD FACSLyric within ≤6h of staining.
    - Review and approve results; if there is an issue with the data acquisition of a tube, rerun the tube. If there is still an issue, acquire the second replicate. The appropriate CRF must be completed.

Day 2 (Second Stain):

- Stain the specimen in duplicate within ≥26/≤28h of draw using Trucount tubes and complete the CRFs. After staining, store the samples in the dark at room temperature for 7-9h.
  - Acquire the data from one replicate on the IUO BD FACSLyric within 7-9h of staining.
  - Review and approve results; if there is an issue with the data acquisition of a tube, rerun the tube. If there is still an issue, acquire the second replicate. The appropriate CRF must be completed.

Table 3. 6C-Multitest Testing Matrix

6C-MT		Claim 24 AOB/6 AOS	
		AOS (h)	
		0-6	8(7-9)
AOB (h)	0-6	X	NA
	24 (23-25)	X	
	27 (26-28)	NA	X

X Time point
X Claim
X Time point after Claim

Table 4. Study Time Point for 6C-MT Reagent, Age of Blood and Age of Stain

No.	Time Point	Age of Blood (AOB)	Age of Stain (AOS)
1	T0 /T0	Reference	
		≤6h	0-6h
2	T24/T6	23-25h	0-6h
3	T27/T8	26-28h	7-9h

6.5 Data Review and Analysis

After data acquisition, all data from the investigational instrument should be reviewed and gates adjusted, as necessary. Store data electronically and print all reports for the data binders. Export CSV files.

- Review documentation and prepare data packet, to include electronic instrument data files, PDFs, completed CRFs, etc.
- Store data on provided electronic devices and in data binders.
- For submission of data to BD, print outs and CRFs must be scanned into electronic files. Provide data package to BD using the password protected BD format transfer protocol (FTP) site or, if required, by mail.

- Site should submit the entire package of data at the end of the last time point of collection for each specimen.

6.6 Required Training and Proficiency Testing

All study staff participating in the study will be trained in the study design and study procedures. Training will be documented. Each operator must also pass proficiency evaluation. Proficiency testing will involve performing the study workflow, including instrument startup, processing and running of controls and specimens, completing all appropriate paperwork, and submission of data. The data will be compared to the expected criteria, and paperwork (CRFs, etc.) will be evaluated for determination of the operator passing proficiency. Instructions for proficiency testing will be provided and documentation of operator proficiency will be given to the site and filed in the TMF prior to study execution.

6.7 Customer Ease of Use/Usability

The IUO BD FACSLyric System will be evaluated in a minimum of two operators per site after they have completed Proficiency Training and before the start of enrollment. The operators will complete and return the Ease of Use survey to BD.

- Ease of use survey will consist of questions addressing workflow and functionality of the IUO BD FACSLyric System. Each question will have 6 possible answers with a numeric value assigned for analysis. Example of the answers as the following:
  - Extremely easy/satisfied, numeric value = 5
  - Somewhat easy/satisfied, numeric value = 4
  - Neutral, numeric value = 3
  - Slightly difficult/dissatisfied, numeric value = 2
  - Very difficult/dissatisfied, numeric value = 1
  - Not applicable (NA), numeric value = 0

6.8 Additional Site Responsibilities

6.8.1 IUO BD FACSLyric Monthly Maintenance

The site will be responsible for normal monthly maintenance of the IUO BD FACSLyric flow cytometer. On a monthly basis, the "monthly clean procedure" needs to be performed. Instructions are in the Instructions for Use. Documentation of cleaning needs to be sent to BD via the instrument preventive maintenance record.

Also, BD FC beads need to be run every 30 days to set compensation controls in order for study data to be evaluable. Documentation of running the BD FC beads needs to be sent to BD via the instrument preventive maintenance record.

6.9 Communication with BD

As necessary, the site will be required to respond to DCFs and other study related questions in a timely fashion. Delay in responding could result in data being considered non-evaluable.

7.0 DISCONTINUATION OF TESTING

7.1 Discontinuation of study specimens

Post-enrollment, instances may occur that will require the discontinuation of the specimen testing such as:

- If further visual inspection of an enrolled specimen reveals clotting and/or hemolysis that was not initially apparent. This specimen would be unsuitable for study testing or, if results have been generated, these results would not be included in analysis.
- If an enrolled specimen (of sufficient quantity) is broken/spilled, this "quantity not sufficient (QNS)" specimen would require discontinuation of study testing.
- If an enrolled specimen (with adequate time post-draw to enroll for study testing) cannot be stained within the allotted time due to unanticipated testing difficulties, this specimen would require discontinuation of study testing. The decision to discontinue testing of a specimen can be made for each reagent.

7.2 Replacement of Discontinued Specimens

Discontinued specimens will be replaced by additional enrolled specimens.

7.3 Retention of Data from Discontinued Specimens

No data will be collected from specimens after the point of discontinuation, except as needed to follow ongoing adverse events. All study data collected from the specimen up to the point of discontinuation will be recorded on the Case Report Form, entered into the study database, and included in subsequent analyses, as appropriate.

8.0 RISK / BENEFIT ASSESSMENT

8.1 Potential Risks

Site(s) will use only prospectively procured specimens for the execution of this protocol. There are no risks to the patient/subject other than those involved with the collection of a blood draw. In addition, the results produced for this study:

- Will not be used for diagnosis or treatment of the patient/subject;

To reduce the likelihood of any risks to site staff participating in this investigation, all processing and testing of potentially infectious specimens must always be performed according to Standard Precautions, CDC Guidelines, Standard Guidelines, and the participating sites' standard operating procedures and policies.

8.2 Potential Benefits

There are no direct benefits to the subject for their specimens being enrolled in this study.

9.0 ASSESSMENT OF SAFETY AND ADVERSE EVENTS

9.1 Adverse Event Definitions

Adverse Event (AE): Any unfavorable and unintended sign (including an abnormal laboratory finding), symptom or disease in a subject that is temporally associated with the use of an investigational product or participation in an investigation, even if the event is not considered to be related to the study product or procedures.

This includes events not seen at baseline and events that have worsened if present at baseline. The term AE will refer to all adverse events (serious and non-serious) occurring during participation in a study of either investigational devices and/or drugs.

Serious Adverse Event (SAE): An SAE is any AE occurring during study participation that results in any of the following outcomes:

- Death
- Life Threatening (refers to any event in which the subject was at risk of death at the time of the event; it does not refer to an event which hypothetically might have caused death if it were more severe)
- Hospitalization or prolongation of a hospital stay
- Persistent or significant disability or incapacitation (refers to any event which results in a substantial and/or permanent disruption of the subject's ability to carry out normal life functions)
- Required intervention to prevent permanent impairment/damage
- Congenital anomaly/birth defect
- Important medical event that may require intervention to prevent one of the preceding conditions.

Unanticipated Adverse Device Effect (UADE): Any serious adverse effect on health or safety or any life-threatening problem or death caused by, or associated with, a device, if that effect, problem, or death was not previously identified in nature, severity, or degree of incidence in the investigational plan or application (including a supplementary plan or application), or any other unanticipated serious problem associated with a device that relates to the rights, safety, or welfare of subjects. Refer to Protocol Section 8.1 (Potential Risks) for a list of *anticipated* adverse events, signs or symptoms. (21CFR-812.3(s))

9.2 Adverse Event (AE) Management

At each study contact, subjects will be questioned in an open-ended manner regarding any new or worsening undesirable signs or symptoms they may have experienced since the previous contact. Elicited signs and symptoms must be comprehensively documented on the appropriate source documentation.

Each sign, symptom, disease or illness reported must be evaluated by the Investigator or designee to determine if it meets the definition of an Adverse Event.

The clinical course of the event will be followed according to accepted standards of medical practice until the event resolves, stabilizes, or in the opinion of the Investigator, is no longer considered clinically significant. The Investigator must supply the Sponsor with information concerning the follow up and/or resolution of the AE.

9.2.1 Study-Specific Exceptions to AE reporting:

Some signs and symptoms are inherent to the study procedures (such as phlebotomy) are likely to occur transiently for nearly all subjects in this study. The following will not be considered AEs:

- Brief, mild pain, mild bruising, and bleeding at the venipuncture site

The above signs and symptoms will not be reported on the Adverse Event CRF as long as they occur in the same circumstances, extent and severity as described above.

However, these signs and symptoms ***must be reported as AEs*** should any of them occur in such a way that the extent or nature of the experience exceeds that normally associated with the procedure, as judged by the PI, or the event meets the criteria for a Serious Adverse Event (SAE).

9.3 Assessment of Adverse Events (AEs)

All AEs must be assessed for Seriousness, Severity, and Relationship. All AEs, regardless of classification, must be comprehensively documented in the CRF and on the SAE form, if applicable, and reported to BD. This includes AEs related to marketed study products. The following information about the event is to be reported on the AE CRF:

- Seriousness, classified as: Non-Serious or Serious
- Severity, classified as:
  - Mild: Transient symptoms, easily tolerated, no interference with daily activities
  - Moderate: Marked symptoms, moderate interference with daily activities, tolerable
  - Marked: Considerable interference with daily activities, intolerable
- Relationship, to the study product or study procedures:
  - Probable: Good reasons and sufficient documentation to assume causal relationship
  - Possible: Causal relationship is likely and cannot be excluded.
  - Unlikely: The event is most likely related to an etiology other than the study treatment
  - Not Related: Clearly unrelated to study treatment or procedures

In addition, the following should be recorded for each AE:

- Action(s) taken to remedy the AE, including change in study treatment or participation, or medical/surgical treatments
- Duration of the AE from onset through resolution, as applicable
- Cause (including suspected product/procedure and/or other cause)
- Outcome of the event, including resolution and sequelae, as applicable

9.4 Additional procedures for Assessing & Reporting Serious Adverse Events (SAE)

SAE criteria are specified in Section 9.1. All SAEs must also be assessed by the Investigator and Sponsor Medical Monitor to determine whether an SAE is expected or unexpected. An adverse event will be considered unexpected or unanticipated if the nature, severity or frequency of the event is not consistent with the risk information previously described in the protocol, Informed Consent, or Investigator's Brochure (if applicable).

Any adverse event meeting the criteria for 'Serious', regardless of the Investigator's opinion of expectedness or relationship to the study product, must be reported to BD within 24 hours. The Investigator or designee must report the event by telephone or email to the Study Monitor. In addition to reporting the SAE to the Study Monitor, the Investigator must also submit a completed SAE form to the BD Trial Safety Dept. via fax or email listed below within 24 hours of receipt of the information.

- Safety Fax Line: (US) 1-201-847-5688
- Safety Email: BD_Trial_Safety@BD.com

9.4.1 Reporting Obligations to IRB/EC and Health Authorities

The Investigator must report any adverse events which are serious, unanticipated/unexpected and probably or possibly related to the study product or procedures to the reviewing IRB/EC. This report

must be submitted as soon as possible, but in no event later than 10 working days after the Investigator first learns of the event.

The Investigator may also have additional responsibilities for AE reporting to their governing Health Authority which they are responsible for identifying and fulfilling.

The Sponsor will provide results of any evaluation of an unanticipated/unexpected adverse device effect to appropriate Health Authorities, to all Investigators, and to all reviewing IRB/ECs within 10 working days after the Sponsor is notified of the event. If the Investigator wishes to assume responsibility for filing reports of evaluation results to their own IRB/EC in lieu of the Sponsor, they must notify the Sponsor in writing of this preference and must retain evidence of their compliance with this requirement.

BD will comply with all other Sponsor safety reporting requirements and timelines for other entities (e.g., Data Safety Monitoring Boards) and local health authorities in other countries where this study or other studies with the same product are being conducted, in compliance with study procedures and applicable local regulatory requirements and BD Standard Operating Procedures.

10.0 INCIDENTS

A Clinical Study Incident is defined as any problem or issue involving the investigational product(s), reference methods, associated procedures or equipment, or represents a product-related injury (or potential for injury) to study subjects or personnel as a result of execution of this protocol. Clinical Study Incidents may adversely (or potentially adversely) affect human safety, the integrity of the evaluation data, or the operation of devices or systems, and warrant prompt attention.

Incidents involving injury to study subjects will also be reported as Adverse Events (refer to Section 9). Examples of Clinical Study Incidents that are not Adverse Events might be mislabeling or adulteration of the investigational device, equipment or device malfunctions, error's in the device instructions, damage to devices caused by shipping or handling or improper storage, or injury to study personnel due to execution of the protocol. If appropriate, an Incident may also be documented and reported as a protocol deviation.

Study-specific procedures for reporting Incidents, as well as adverse events and protocol deviations, will be provided to the study site prior to study execution. The Monitor should be contacted immediately when site becomes aware of or suspects any defective or malfunctioning product. This includes:

- Products that are involved in Study Incidents,
- Products that are found to be expired, damaged or defective,
- Products that are possibly the cause of an adverse effect, regardless of whether the product was believed to be damaged, defective or malfunctioning.

Such products (whether investigational or marketed) should be segregated and returned with appropriate documentation to the BD address below, unless instructed otherwise by BD. The Study Monitor should be contacted with any questions regarding return of study products. BD will supply mailing kits specifically intended for product contaminated with potentially bio-hazardous material.

11.0 RETURN OR DESTRUCTION OF STUDY PRODUCT

All disposable, used products not failed, damaged or otherwise involved in an Incident or Adverse Event are to be discarded into appropriate waste containers at the investigational site.

Unless instructed otherwise by BD, the Investigator will return all remaining unused or unopened test, reference, and ancillary study products to BD. At the conclusion of the study, and as appropriate during

the course of the study, any products, supplies or BD equipment that are required to be returned will be shipped to BD at the address below, unless instructed otherwise:

Study Manager
2350 Qume Drive
San Jose, CA 65131
(408) 954-2384

11.1 Source Documents

Source data includes all information in original records (and certified copies of original records) of clinical findings, observations, or other activities (in a clinical study) used for the reconstruction and evaluation of the study. Source data are contained in source documents (original records or certified copies) and are used to verify the authenticity of information recorded on the Case Report Form (CRF). Typical source documents include the hospital chart, medical office file, laboratory report, clinician notes, patient record, recorded data from automated instruments or other documentation prepared and maintained by the investigator/staff or ancillary services which contains a record of all observations and other data pertinent to the investigation on a study subject.

The investigator is required to maintain original source documents at the site. Should an original source document (e.g., an instrument printout, direct entry CRF) need to be forwarded to BD for data entry, the site must retain a clearly designated certified copy. The Study Monitor will confirm that procedures for copy certification have been established at the site prior to transmittal of any original source documents.

11.2 Case Report Forms (CRF)

The case report forms (CRF) will be provided by the Sponsor. The term "CRF" as used in this protocol may refer to traditional paper CRFs, or electronic case report forms for electronic data capture (EDC), as determined by the Sponsor.

The Investigator may delegate CRF completion to study personnel. However, the Sponsor must be apprised in writing of the name of such persons and the scope of their authority. The Principal Investigator or designee is obligated to review each CRF page and sign or initial the indicated pages using ink or for EDC, an electronic signature. An individual record will be kept for each subject that provided informed consent.

All entries to a paper CRF should be made clearly in black or dark blue indelible ballpoint pen to ensure the legibility of self-copying or photocopied pages. Corrections are made by placing a single horizontal line through the incorrect entry, so that the original entry can still be seen, and placing the revised entry beside it. The revised entry must be initialed and dated by a member of the Investigator's research team authorized to make CRF entries. Correction fluid must not be used.

CRF entries will be compared to source documents by the study monitor or designated personnel. Unless specified otherwise, all information on the CRFs must be traceable to original source documents.

11.2.1 CRF as Source Document

Some study data will be collected directly on CRFs and there will be no matching source from which to verify the entry. As a result, these CRFs serve as original source documents.

11.2.2 CRF/Data Transmittal

CRF and other data should be transmitted to the sponsor within one week of the testing date. Instructions for CRF and Data Transmittal will be provided to the Investigator at Study Initiation.

Specific procedures may be described in a study-specific Monitoring Plan. BD will provide access and instructions to a secure File Transfer Protocol (FTP) site to accomplish this transfer of data.

11.3 Electronic Source Data (optional)

Electronic files generated from study instruments contain source data. The site must maintain electronic files of all these data on storage devices (CDs, USB drives, etc.) provided by the Sponsor.

Some electronic source data will need to be transmitted to the Sponsor within one week of the testing date. Instructions for Data Transmittal will be provided to the Investigator at study initiation.

Specific procedures may be described in a study-specific Data Management Plan.

11.4 Data Management and Storage

Data Management will be performed by the Sponsor. Data from completed CRFs will be entered into a controlled database and the database verified for accuracy against the CRFs, when applicable. If electronic data capture is utilized, the electronic records entered at the site will be entered directly into the controlled database. Data security is ensured through password protection, limited access, audit trails, and regular backups of the data. During the data management process there will be edit checks run against the data that is entered in the database. This may result in Data Clarification Forms (DCF) being generated to provide to the site for clarification of data points entered on the CRF. This process will be defined in the training materials provided to the site at initiation. Upon completion of the study and verification of data, data will be screened for accuracy and completeness, after which the database will be locked from any additional changes. A copy of the locked database will be provided to the BD Corporate Statistics Department for statistical analysis.

12.0 STATISTICAL METHODS

12.1 Sample Size Determination

There will be a minimum of 90 prospectively procured specimens that provide valid results from stability testing for each reagent (Multitest 6C TBNK reagent, and Multitest IMK kit reagents). It is anticipated that enrollment may be up to 500 specimens required to fulfill the bin requirements as discussed in this Statistical Methods section. Depending on the volume of the venous whole blood specimen enrolled, one or more of the assays may be tested on that specimen.

12.2 Data Evaluability

Data will be considered evaluable if all the following conditions are met:

- Instrument maintenance is up-to-date.
- Adequate controls have been run and are acceptable.
- Evaluable data has been produced from testing of an evaluable specimen (has met inclusion criteria).
- Time Zero has been obtained for the specimen.
- Sample replicates of time point were acquired within the allotted times for the assay (Age of Blood and Age of Stain).
- Results are within the reportable range of the assay.
- Data packets are complete and all forms are complete.
- Documentation is complete.

Discrepancies in source documents can result in the data being considered non-evaluable.

12.3 Statistical Methods

12.3.1 General Statistical Consideration

All statistical analyses will be performed using commercially available statistical software. Adequate source document verification and/or audit activities will be utilized to assure the validity of the stability evaluation conclusions. Analysis shall be performed based on methods described in the Statistical Analysis Plan. All related and resulting reports, documents and data shall be produced and maintained in such a way as to ensure their control and the protection of subject privacy as far as is reasonably practicable. Data files and analytic reports will be archived according to requisite regulatory standards.

12.3.2 Stability Statistical Methods

This study will be performed in accordance with CLSI guidelines.

The minimum number of EDTA venous whole blood specimens that provide valid study results (i.e., meeting all inclusion criteria and protocol requirements) is 90 from adolescent/adult subjects* to satisfy enrollment by the bins shown below. In order to fill bins, up to 15% of total specimens may also be manipulated. Specimens will be binned by CD4 measurement of the "Reference" result (see below). For each reagent (, 4C-Multitest and 6C-Multitest), the desired minimum number of enrolled venous whole blood specimens for AbsCD4 and %CD4 bins is as listed below in Table 5:

Table 5. Minimum Required WBC Specimens

	**Bins (cells/µL)**	**Total # Desired***	**Min**
**AbsCD4**	1) 0 ≤ CD4 < 200	30	30
	2) 200 ≤ CD4 < 500	20	15-20
	3) 500 ≤ CD4 < 1000	20	15-20
	4) 1000 ≤ CD4 ≤ 4500	20	15-20
**%CD4**	**Bins (%CD4)**	**Total # Desired***	**Min**
	1) 0% ≤ CD4% < 20%	13	10
	2) 20% ≤ CD4% < 35%	14	10
	3) 35% ≤ CD4% ≤ 100%	13	10

* To satisfy enrollment by bin, specimens from pediatric subjects may be included, since relative lymphocytosis and the higher CD4+ lymphocyte counts are normal in children less than 5 years of age[1].

For each specimen, the "Reference" result is the value at time zero (i.e., the result from fresh blood/fresh stain) and the "Test" results correspond to the values obtained at the subsequent testing at different time points (2, 3, etc.) The difference between the Test and Reference results will be calculated for each specimen, and pooled across all specimens to produce the mean difference for each time point with 95% confidence interval. The 95% confidence of the mean % difference has to be within the above acceptance criteria (in Section 3.3).

.

12.4 Demographics/Other descriptive information

N/A. This study will not generate demographics or other descriptive information.

12.5 Interim analysis

Interim analysis of the study data may be performed as needed.

13.0 QUALITY CONTROL AND ASSURANCE

13.1 Accountability of Study Products

Investigational study products will only be released for use to Investigators who have obtained written IRB/EC approval (as required) for participation in this study, who have completed all required study documentation, and who have been qualified by the Sponsor. Investigators must maintain control over all study products, and ensure they are used in accordance with this protocol. Failure to do so may result in the Sponsor suspending or terminating the study at the Investigator's site.

The Investigator will ensure that study products are only dispensed to subjects (or used for specimens) properly enrolled in the study. The Investigator must maintain records of receipt, disposition, return and/or destruction of all study products. All investigational study products released to the site must be accounted for at the unit level prior to study close out, regardless of disposition. The Study Monitor will regularly review all records regarding study product accountability.

The Sponsor will maintain records that document the shipment, receipt, disposition, return and/or destruction of study products.

13.2 Monitoring

BD, the study sponsor, will designate trained and qualified personnel to monitor the progress of this clinical study in accordance with BD Monitoring SOPs and the study-specific Monitoring Plan. A pre-study site qualification visit will be conducted to assess the adequacy of the site facilities and staff with respect to study requirements.

Prior to study start, a study initiation visit will be conducted to provide training to site staff with regard to the protocol, the completion of study documentation and Case Report Forms (CRFs), the monitoring schedule, and all regulatory requirements. During the study, routine monitoring visits will be conducted (as applicable based on the duration of the study) to assure the site continues to adhere to the protocol, the investigator agreement, and regulations regarding conduct of clinical studies. Assessments will be made regarding the subjects' protection and safety, when relevant, as well as the quality, completeness, and integrity of the data. The Study Monitor will assist the investigative site with query resolution and will perform site close-out activities once all queries have been resolved.

Additional visits may be carried out depending upon site activity and performance. The Investigator must agree to the inspection of all study related records and give direct access to source documents for verification of data on CRFs.

The Investigator is responsible for ensuring that any site-owned equipment required for use in the study is properly installed and maintained (e.g., inspected, calibrated, alarmed). Documentation of equipment maintenance procedures must be available for review by the Monitor.

13.3 Audits and Inspections

If the study is selected for audit by the Sponsor or if there is an inspection by the appropriate Health Authorities, then the Investigator and his team will make themselves available during the visit. The Investigator must agree to the inspection of all study related records and give the auditor/inspector direct access to source documents for verification of data on CRFs. The subject's anonymity must be safeguarded and data checked during the audit remain confidential.

As soon as the Investigator is aware of an upcoming inspection/audit by the Health Authorities, he/she will promptly inform BD. As agreed with the Investigator, BD personnel may be present at the site during the inspection.

13.4 Protocol Deviations

Protocol deviations are not permitted and should be implemented prospectively as a protocol amendment whenever practical or appropriate, unless required to protect the safety and well-being of the subject. The Investigator must notify the Sponsor immediately of any such deviation resulting from the need to protect a subject.

Protocol deviations (other than those required to protect the safety and well-being of a subject) may impact the evaluability of study data, and may place subjects at risk. If the Investigator or their staff inadvertently deviates from the study plan, the Investigator should implement appropriate corrective and preventive procedures, and should notify the Sponsor at their earliest convenience. Significant deviations may also need to be reported to the IRB/EC and local health authority.

The Study Monitor will evaluate records of study conduct at the site to identify any deviations, and will also report them to the Sponsor. Upon evaluation by the Sponsor, actions may be required to prevent additional deviations, such as retraining of the site, implementation of additional site procedures, and more frequent monitoring. If these steps fail, more serious measures, up to and including termination of the site and withdrawal of study product may be necessary.

14.0 ETHICAL AND REGULATORY STANDARDS

14.1 IRB/EC

An appropriate IRB/EC must review this protocol, the Informed Consent Form (if applicable), and any other supporting study documents which affect subject or study personnel safety, prior to study initiation at an investigational site. No study product will be released for use by a site, and no investigational site may begin the study until the IRB/EC has given its written approval, signed by the IRB/EC chairperson or authorized personnel, and a copy of the approval letter and the approved Informed Consent Form (if applicable) has been provided to the Sponsor.

14.2 Informed Consent

Prospectively collected specimens enrolled in the study will be by Informed Consent. All of the subject's personal health information will be removed prior to enrollment and they will be given a BD Study number.

14.3 Confidentiality of Data

Subject confidentiality is strictly held in trust by the participating investigators, their staff, and BD and their agents. This confidentiality is extended to cover testing of biological samples in addition to the clinical information relating to participating subjects. Subject confidentiality and anonymity will be maintained at

all times by removal of all identifiers from any data, clinical samples or documentation submitted for this study.

Any data collected meeting the definition of PHI will be collected and maintained using the designated authorizations and following all privacy procedures as specified in the applicable health authority regulations.

BD will maintain the security and confidentiality of all clinical study data sent to BD. BD clinical study databases will not be shared with any third party without the express written consent of the Principal Investigator and/or Site.

The Study Monitor or other authorized representatives of BD may inspect all documents and records required to be maintained by the Investigator. The Site will permit access to such records. BD and the Site may be required to provide regulatory agencies access to clinical study data and records, as well as source documents.

All other agreements as to confidentiality by BD, the Principal Investigator, and the Site may be found in the Confidential Disclosure Agreement and the Clinical Trial Agreement.

14.4 Protocol Modifications

Amendments to the protocol will not be implemented without agreement from the Sponsor and prior submission to and written approval from the governing IRB/EC (as applicable), except when necessary to eliminate an immediate hazard to the subject. Notice of an emergency modification shall be given to the Sponsor and the reviewing IRB/EC as soon as possible, but in no event later than 5 working days after the emergency occurred. Protocol amendments may affect Informed Consent Forms for current and future subjects.

Minor changes to the protocol, such as correction of typographical errors or changes in personnel names (other than the PI) or contact information will be processed as administrative changes. Administrative changes will be submitted to the governing IRB/EC but implementation of the administrative change may proceed without prior IRB/EC approval, unless so required by the IRB/EC or site SOPs.

14.5 Study Discontinuation

BD reserves the right to temporarily suspend or prematurely discontinue the study at a single site or at all sites at any time and for any reason. If such action is taken, BD will discuss the reasons with all Investigators (the Investigator). If the study is terminated or suspended due to safety reasons, the sponsor will inform the health authorities as required, and provide the reason(s) for the action. Investigator(s) must inform their IRB/EC promptly and provide the reason(s) for the suspension or termination.

14.6 Clinical Study Registration

In compliance with Title VIII of Public Law 110-85, known as FDA Amendments Act of 2007 (FDAAA), BD will register all applicable studies and disclose study results in a publicly accessible database, e.g. the ClinicalTrials.gov web site. Applicable studies will be registered no later than 21 days after commencing enrollment. Study results for applicable studies will be posted to the website within 12 months of the last subject visit for collection of primary outcome data, or after health authority approval for previously unapproved devices. BD has responsibility for determining whether this study qualifies as an "applicable" study under the law, and if so, will take responsibility for registration and disclosure as required by law.

14.7 Publication of Results

BD believes that results of applicable clinical studies of our products should be published in peer-reviewed literature in a timely, accurate, complete and balanced manner, regardless of study outcomes. BD is committed to making information public whenever it relates to the safety and efficacy of its marketed products.

Should this study be considered an "applicable study," any formal presentation or publication of data collected from this study will be considered as a joint publication by the investigator(s) and the appropriate personnel of BD. Authorship will be based on generally accepted criteria of the ICMJE (International Committee of Medical Journal Editors) and determined by mutual agreement. For multi-center studies, it is mandatory that the first publication is based on data from all centers, analyzed as stipulated in the protocol by BD statisticians, and not based on data from single sites or a subset of sites. Investigators participating in multi-center studies agree not to present data gathered from one center or a small group of centers before the full, initial publication, unless formally agreed to by all other investigators and BD (the sole exception being an unanticipated adverse event that is product-related and which might have clinically significant safety implications for a marketed product or a class of products).

BD must receive copies of any intended communication in advance of publication as specified in the Clinical Study Agreement. In a timely manner, BD will review the communications for accuracy (thus avoiding potential discrepancies with submissions to health authorities), verify that confidential information is not being inadvertently divulged and to provide any relevant supplementary information to the investigators.

14.8 Record Retention

If the Principal Investigator or Clinical Center withdraws from the responsibility of keeping the study records, custody must be transferred to a person or entity who will accept the responsibility. BD must be notified in writing of the name and address of the new custodian.

Federal regulations require that a copy of all essential study documents (e.g., IRB/EC approvals, signed informed consent forms, source documents, CRF copies, safety reports, test article dispensing records, etc.), must be retained in the files of the responsible Investigator for a minimum of 2 years following notification by BD that all investigations are completed, terminated, or discontinued, or that the FDA has approved the application (21 CFR 812.140).

15.0 BIBLIOGRAPHY/REFERENCES

E6, International Conference on Harmonization: Good Clinical Practice: Consolidated (Published in the Federal Register May 9, 1997).

CLSI. Evaluation of Stability if In Vitro Diagnostic Reagents; Approved Guideline, CLSI document EP25-A. Wayne, PA: Clinical Laboratory Standards Institute; 2009.

PROTOCOL REVISION HISTORY

Version #	Rationale for Change	Section or Page affected	Description of change
1.0	New Protocol		
1.1	Clarify definition for Age of Stain	Section 3.1 Study Design	The Age of Stain should read as the end of the lysing step until the time of specimen acquisition.
1.1	Clerical error for time point T51/T26 had range for AOS as 26-28 and it should be 25-27.	Section 6.4.1 Multitest IMK kit workflow	AOS range is stated as 26-28 and it should be 25-27
2.0	The word “external” when describing the study sites should not be used as the descriptive. The primary objective requires at least 2 study sites.	Section 2.1 Primary Objectives	The word “external” when describing study sites being used in the primary objectives has been removed.
