# Supplementary material for: Accurate and reproducible enumeration of T-, B-, and NK lymphocytes using the BD FACSLyric 10-color system: A multisite clinical evaluation
Source: PLoS One. 2019 Jan 28;14(1):e0211207. doi: 10.1371/journal.pone.0211207 (PMC6349332; doi:10.1371/journal.pone.0211207)
Supplement: S1 Table — (DOCX) [file pone.0211207.s009.docx]

**S1 Table. Reference intervals by gender.**

| **Reagent and T-, B-, and NK Lymphocytes** | | **Male**  **mean** | **Female mean** | **Statistically significant difference** | |
| --- | --- | --- | --- | --- | --- |
|  |  |  |  | **Harris Boyd** | **Anova F** |
| **IMK  M^k^= 65  F^l^= 65** | **AbsCD3 Ave** | **1424.15** | **1687.59** | **Yes** | **0.00111** |
|  | **AbsCD3 T1** | **1418.43** | **1684.14** | **Yes** | **0.00094** |
|  | **AbsCD3 T2** | **1429.91** | **1690.97** | **Yes** | **0.00145** |
|  | **AbsCD4** | **892.09** | **1114.91** | **Yes** | **0.00003** |
|  | **AbsCD8** | 494.82 | 533.57 | No | 0.41126 |
|  | **AbsCD16^+^CD56** | 283.03 | 302.43 | No | 0.34594 |
|  | **AbsCD19** | 301.52 | 260.55 | No | 0.14558 |
|  | **%CD3 Ave** | **69.89** | **73.88** | **Yes** | **0.00159** |
|  | **%CD3 T1** | **69.98** | **74.02** | **Yes** | **0.00160** |
|  | **%CD3 T2** | **69.80** | **73.74** | **Yes** | **0.00170** |
|  | **%CD4** | **44.03** | **48.99** | **Yes** | **0.00076** |
|  | **%CD8** | 23.63 | 22.87 | No | 0.58057 |
|  | **%CD16^+^CD56** | **15.02** | **11.48** | **Yes** | **0.00110** |
|  | **%CD19** | 13.87 | 13.52 | No | 0.67238 |
| **TBNK  M^k^= 65  F^l^= 69** | **AbsCD3** | **1413.60** | **1690.91** | **Yes** | **0.00041** |
|  | **AbsCD4** | **886.17** | **1112.58** | **Yes** | **0.00002** |
|  | **AbsCD8** | 481.52 | 528.97 | No | 0.28271 |
|  | **AbsCD19** | 283.35 | 312.32 | No | 0.18350 |
|  | **AbsCD16^+^CD56** | 308.31 | 268.67 | No | 0.13834 |
|  | **%CD3** | **70.06** | **73.73** | **Yes** | **0.00293** |
|  | **%CD4** | **44.30** | **48.82** | **Yes** | **0.00193** |
|  | **%CD8** | 23.44 | 22.69 | No | 0.58024 |
|  | **%CD16^+^CD56** | **15.40** | **11.82** | **Yes** | **0.00069** |
|  | **%CD19** | 13.82 | 13.86 | No | 0.96453 |

^k^male; ^l^female
